# OsNBL1, a Multi-Organelle Localized Protein, Plays Essential Roles in Rice Senescence, Disease Resistance, and Salt Tolerance

**DOI:** 10.1186/s12284-020-00450-z

**Published:** 2021-01-09

**Authors:** Xiaosheng Zhao, Tianbo Zhang, Huijing Feng, Tiancheng Qiu, Zichao Li, Jun Yang, You-Liang Peng, Wensheng Zhao

**Affiliations:** 1 State Key Laboratory of Agrobiotechnology, MOA Key Lab of Pest Monitoring and Green Management, Department of Plant Pathology, China Agricultural University, Beijing, 100193 China; 2 Key Laboratory of Crop Heterosis and Utilization, the Ministry of Education/Key Laboratory of Crop Genetic Improvement, Beijing Municipality/ College of Agronomy and Biotechnology, China Agricultural University, Beijing, 100193 China

**Keywords:** Premature senescence, Disease resistance, Salt tolerance, *Oryza sativa*, OsNBL1, OsClpP6

## Abstract

**Background:**

Plant senescence is a complicated process involving multiple regulations, such as temperature, light, reactive oxygen species (ROS), endogenous hormone levels, and diseases. Although many such genes have been characterized to understand the process of leaf senescence, there still remain many unknowns, and many more genes need to be characterized.

**Results:**

We identified a rice mutant *nbl1* with a premature leaf senescence phenotype. The causative gene, *OsNBL1,* encodes a small protein with 94 amino acids, which is conserved in monocot, as well as dicot plants. Disruption of *OsNBL1* resulted in accelerated dark-induced leaf senescence, accompanied by a reduction in chlorophyll content and up-regulation of several senescence-associated genes. Notably, the *nbl1* mutant was more susceptible to rice blast and bacterial blight but more tolerant to sodium chloride. Several salt-induced genes, including *HAK*1, *HAK*5, and three *SNAC* genes, were also up-regulated in the *nbl1* mutant. Additionally, the *nbl1* mutant was more sensitive to salicylic acid. Plants overexpressing *OsNBL1* showed delayed dark-induced senescence, consistent with a higher chlorophyll content compared to wild-type plants. However, the overexpression plants were indistinguishable from the wild-types for resistance to the rice blast disease. OsNBL1 is a multi-organelle localized protein and interacts with OsClpP6, which is associated with senescence.

**Conclusions:**

We described a novel leaf senescence mutant *nbl1* in rice*.* It is showed that OsNBL1, a multi-organelle localized protein which interacts with a plastidic caseinolytic protease OsClpP6, is essential for controlling leaf senescence, disease resistance, and salt tolerance.

**Supplementary Information:**

The online version contains supplementary material available at 10.1186/s12284-020-00450-z.

## Background

Leaf senescence, a natural process involving programmed cell death in plant development, facilitates nutrient remobilization and plays a crucial role in crop yield (Lim et al., 2007; Wu et al., 2012b). The initiation and progression of leaf senescence is a complicated process, controlled by a precise molecular regulatory network. Revealing the mechanism of leaf senescence would provide a way to improve agricultural traits of crop plants (Lim et al., 2007; Schippers, 2015). Symptoms of leaf senescence include the degradation of chlorophyll and proteins, hydrolysis of lipids, and DNA damage (Lim et al. 2007).

As the final stage of leaf development, leaf senescence is basically governed by developmental age. However, leaf senescence is also induced by various internal and environmental signals, including phytohormones and reproduction, and external factors, such as darkness, UV-B or ozone, nutrient limitation, heat or cold, drought, high salinity, and pathogen attack (Lim et al., 2007; Woo et al., 2013). A typical characteristic of leaf senescence is programmed cell death (PCD). When leaf senescence is initiated, the contents of phytohormones, such as ethylene (ET), jasmonates (JA), salicylic acid (SA), abscisic acid (ABA), gibberellic acid (GA), and cytokinin (CK), are changed, so that they play roles in regulating leaf senescence (Lim et al., 2007; Jibran et al., 2013; Wang et al., 2020; Lim et al., 2020). In addition, in most cases, reactive oxygen species (ROS) accumulate as leaves age and function as positive players in leaf senescence (Wu et al. 2012a; Guo et al., 2017). As environmental stress, pathogen attacks usually induce plants to initiate an innate immune system response that is broadly divided into pathogen-associated molecular pattern (PAMP)-triggered immunity (PTI) and effector-triggered immunity (ETI; Jones & Dangl, 2006). ETI is usually accompanied by a form of pathogen induced PCD (pPCD), displaying a hypersensitive response (HR). In plant-pathogen interactions, pPCD is an effective strategy to limit pathogen spread, especially for biotrophic pathogens (Glazebrook, 2005).

Phytohormones are important internal signals that regulate plant development and senescence. Salicylic acid (SA) is a key phytohormone involved in age-dependent leaf senescence (Lim et al., 2007; Zhang et al., 2013; Guo, et al., 2017; Zhang et al., 2017). A higher concentration of endogenous SA, which promotes the expression of several senescence-associated genes (SAGs), is measured in the senescent leaves of Arabidopsis (Morris et al., 2000; Lim et al., 2007; Khan et al., 2014). An interesting discovery deriving from genome-wide transcriptome analysis indicated that the differentially expressed genes (DEGs) induced by exogenous SA treatment were similar with that in leaf age-dependent senescence (Lim et al., 2007). Moreover, SA accumulation in several mutants, such as *ftsh4–4*, *atg3*, *atg5*, *pat14,* and s3h, involves AAA-protease, autophagy, PROTEIN S-ACYL TRANSFERASE14, and SA3-HYDROXYLASE, all of which exhibit significant leaf premature senescence, while expression of the *nahG* gene in the mutants led to a decrease in the SA level and delayed leaf senescence (Yoshimoto et al., 2009; Zhang et al., 2013; Zhao et al., 2016; Zhang et al., 2017). Additionally, SA biosynthetic pathway defective mutant *sid2* and SA responsive factor mutants *npr1* or *pad4* displayed reduced expression of *SAGs* and delayed senescence phenotypes (Morris et al., 2000; Lim et al., 2007; Guo, et al., 2017), suggesting that SA plays a positive role in leaf aging.

Plastidic caseinolytic proteases (Clp) are a class of important enzymes controlling the degradation of many plastidic proteins and playing an essential role in chloroplast function (Adam et al., 2006; Nishimura et al., 2017). In higher plants, the Clp proteolytic system is expansive, comprising five ClpPs (Ser-type proteases), four ClpRs (nonproteolytic proteins), three ClpCs (Clp AAA+ chaperones), two ClpTs (plant-specific accessory proteins), and a ClpS (adaptor protein) (Sakamoto, 2006; Nishimura & van Wijk, 2015; Williams et al., 2019). Furthermore, the Clp protease complex consists of two subcomplexes; one is an R ring formed by ClpP1 and ClpR1 to ClpR4, and the other is a P ring containing ClpP3 to ClpP6. The ClpCs and ClpS have been shown to connect the degraded substrate proteins with a core complex, and the ClpTs are likely to control substrate protein selection or subcomplex binding (Peltier et al., 2004; Olinares et al., 2011a, 2011b; Sjögren & Clarke, 2011; Bruch et al., 2012). In *Arabidopsis*, several studies have reported that Clps regulate chloroplast biogenesis and plant development. The *clpp4* and *clpp5* mutants have been shown to block embryogenesis and be lethal, while *clpp3* mutants are lethal at the seedling stage in soil. Antisense transgenic plants with partial down-regulation of *CLPP4* and *CLPP6* display a retardation in growth and development and plant chlorosis (Sjögren et al., 2006; Zheng et al., 2006; Olinares et al., 2011b; Kim et al., 2013). An extensive analysis of the CLPR family showed that the *clpR1* knockout mutant displays pale-green plants (Koussevitzky et al.*,* 2007) that can be rescued by overexpression of *CLPR3* (Kim et al., 2009); null mutants of *CLPR2* and *CLPR4* are seedling lethal, and *CLPP5* null mutants are embryo lethal (Kim et al., 2009). The loss of function in ClpC1 leads to reduced chloroplast development and lower photosynthetic activity, which results in leaf chlorosis and arrested *growth (Sjögren* et al.*,* 2004*;* *Zhang* et al.*,* 2018). However, the knockout mutant of another Clp AAA+ chaperone protein, CLPC2, exhibited no major visible phenotype (Constan et al.*,* 2004*;* Park & Rodermel, 2004), while the double mutants of ClpC1 and ClpC2 showed an embryogenesis block in plant development (Kovacheva et al.*,* 2007). In rice, the vyl mutant, also known as clpP6, displayed reduced chlorophyll content, abnormal chloroplast ultrastructure, and alternate expression of chloroplast development and photosynthesis-related genes, resulting in chlorotic leaves and dwarf plants (Dong et al., 2013). These studies have shown that Clps play an essential role in the function of chloroplasts and degradation of chlorophyll, which are closely related to leaf senescence.

In this work, we isolated rice T-DNA mutant *nbl1*, which exhibited retarded growth, leaf senescence, and dark-accelerated senescence, accompanied by up-regulation of several senescence-associated genes and six dark-induced genes. Furthermore, *nbl1* displayed enhanced salt tolerance, and RT-qPCR analysis showed that salt-tolerance genes were constitutively activated in *nbl1*. By TAIL-PCR, we obtained the flanking sequence of *OsNBL1*, which encodes an unknown function protein with a transmembrane domain. Confocal microscopic observation implied that OsNBL1 was a multi-organelle localized protein. Y2H and LCI assays showed that OsNBL1 could interact with a Clp protein, OsClpP6. Interestingly, mutation of OsNBL1 in rice accelerated dark-induced senescence and reduced resistance to *Magnaporthe oryzae* and *Xoo*; however, overexpression plants of OsNBL1 showed no effect on disease resistance, while dark-induced senescence was delayed. Our results revealed that OsNBL1 is a novel regulator of leaf senescence, salt tolerance, and plant immunity.

## Results

### Characterization of the Rice Senescence Mutant *nbl1*

**Fig. 1:**
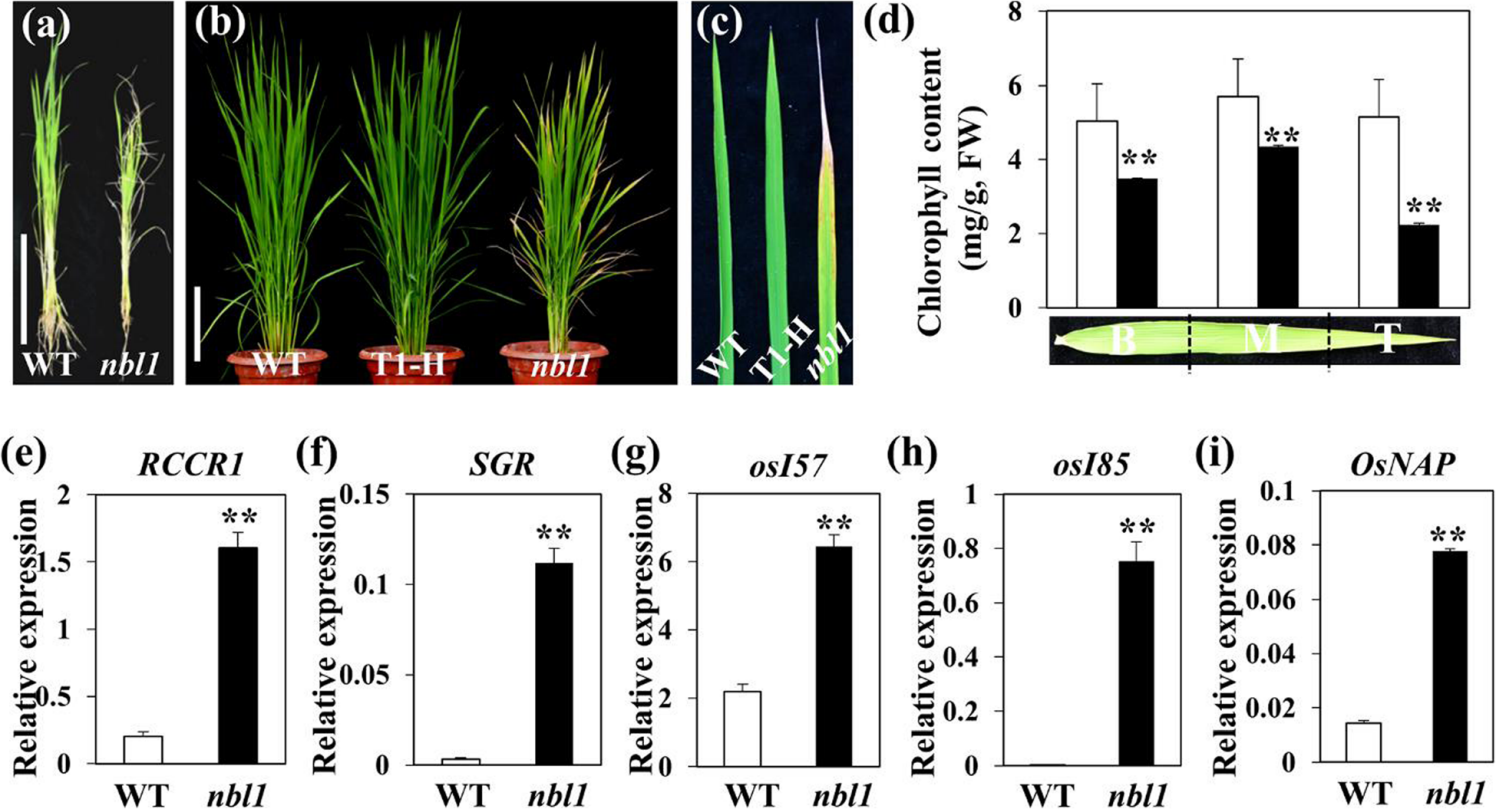
The phenotype of the rice leaf senescence mutant *nbl1*. **a** The picture of *nbl1* mutant and wild-type (WT) plants grown in greenhouse at seedling stage. Scale bar = 15 cm. (b-c) The whole plants (**b**) and flag leaves (**c**) of *nbl1*, T1-H and WT plants grown under paddy filed were photographed at tillering stage. T1-H indicates the heterologous *nbl1* offspring. Scale bar = 20 cm. **d** Flag leaves of *nbl1* and WT plants grown under paddy filed at heading stage were divided into base (B), middle (M), and top (T), and the three sections of leaves were used to determine total chlorophyll content, respectively. Data are means ± SD (*n*=3). **P < 0.01 (Student’s *t*-test). **e**-**i** Transcription levels of five senescence-associated maker genes were analyzed by RT-qPCR and normalized to that of the *OsACTIN1* gene. Total RNA was extracted from the leaves of *nbl1* and WT plants at seedling stage. Data represent means ± SD (*n*=3). **P < 0.01 (Student’s *t*-test)

The mutant *nbl1* (Nature Blight Leaf 1) was identified from a T1 transgenic line of Geng (japonica) rice cv. ‘Aichiasahi’. Under paddy field (Beijing) conditions, *nbl1* plants showed severely retarded development. At the seedling stage, the *nbl1* mutant showed delayed growth, and lower leaves had distinct senescence compared with the wild type. At the tillering stage, the mutant displayed whitish and yellowish leaf tips (Fig. 1a-c), which are hallmarks of early senescence (Li et al., 2014). All five upper leaves of the *nbl1* mutant exhibited significantly accelerated leaf senescence at the heading stage. At the heading stage, flag leaves were divided into three sections, base (B), middle (M), and top (T), for measuring chlorophyll content. The data showed that in each section of the flag leaves of the *nbl1* plant, chlorophyll content was significantly lower than that of wild-type plants (Fig. 1d), demonstrating that the mutant underwent the leaf senescence process earlier than wild-type plants. Moreover, we measured the expression of two chlorophyll degradation-related genes (CDGs), *stay-green* (*SGR*) and *red chlorophyll catabolite reductase 1* (*RCCR1*), and two other SAGs, *Os157* and *Os185,* in the fully expanded leaves of the *nbl1* mutant and wild-type plants. We found that these four genes were significantly upregulated in the *nbl1* mutant compared to wild-type plants (Fig. 1e-h). *OsNAP* is a marker gene of senescence onset in rice that positively regulates leaf senescence by directly targeting genes related to chlorophyll degradation, nutrient transport, and other genes associated with senescence (Liang et al., 2014). The expression of *OsNAP* was significantly higher in the *nbl1* mutant than in wild-type plants (Fig. 1i). These results demonstrated that the leaf senescence process was accelerated significantly in the *nbl1* mutant.

Leaf senescence usually influences various agronomic traits. We found that the height of *nbl1* plants was much shorter than wild-type plants (Fig. S1). The length of each internode of *nbl1* and wild-type plants at the mature stage was measured to investigate the height difference in more detail. Interestingly, we found that *nbl1* plants not only showed shorter internodes but also had one less internode than wild-type plants (Fig. S1a). Moreover, the *nbl1* plants displayed shriveled grain, fewer tillers, a lower seed setting rate, and lower 100-grain weight (Fig. S1b-e).

### The *nbl1* Mutant Showed a Dark-Accelerated Senescence Phenotype

**Fig. 2:**
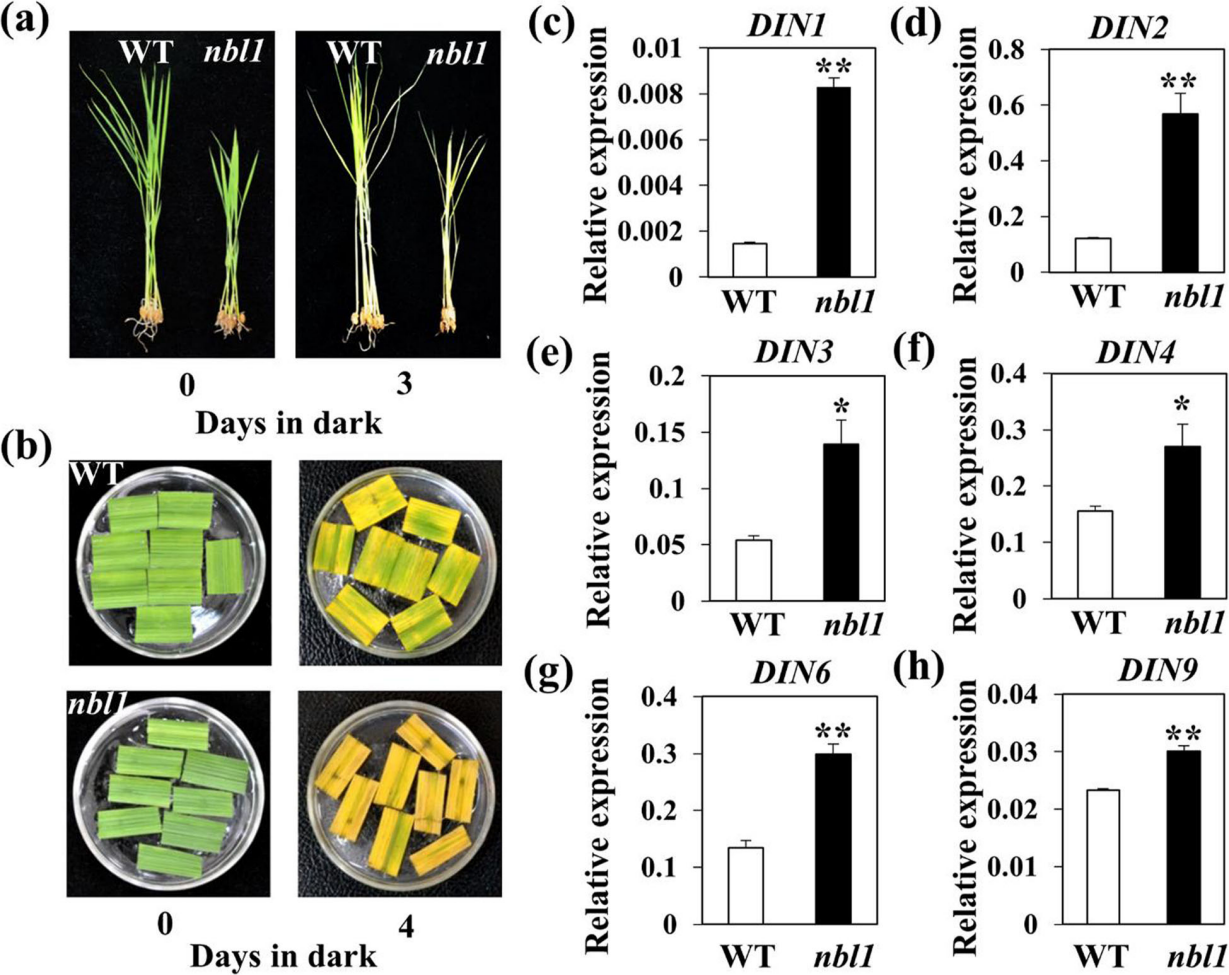
The *nbl1* mutant exhibits accelerated leaf senescence under dark treatment. **a**-**b** Seedlings (**a**) and detached leaves (**b**) of *nbl1* and WT plants were treated with complete darkness at 28 °C. Detached leaves were taken from according rice flag leaves at heading stage, and soaked in buffer solution (3 mM MES, pH 5.8) during darkness. Photographed at 3 days or 4 days after dark incubation (DDI). **c**-**h** The expression of six dark-induced genes (*DINs*) in the leaves of *nbl1* and WT plants were analyzed by RT-qPCR at seedling stage. Data were normalized to the expression of the *OsACTIN1* gene. Values are means ± SD (n=3). **P < 0.01 (Student’s *t*-test)

Darkness is used frequently as an effective method to simulate synchronous senescence because it is one of the most powerful known external stimuli for leaf senescence. To investigate how the *nbl1* mutant responded to darkness, three-week-old seedlings grown in pots were placed in the dark for 6 d and the phenotypic change was observed. Before treatment, all plants were green and healthy. After the dark treatment, *nbl1* plants were more yellow than the corresponding controls (Fig. 2a). We also detached the leaves from *nbl1* and wild-type plants at the heading stage; the middle parts of leaves with the same color between *nbl1* and the wild-type were incubated in complete darkness. The leaf segments of *nbl1* yellowed faster than those of wild-type plants, showing a significant difference at 5 days after dark incubation (DDI; Fig. 2b). This was consistent with the visible phenotype, and the *nbl1* mutant had lower chlorophyll (Chl) levels (Fig. 2c). To further investigate the relationship between the *nbl1* phenotype and darkness, we checked the expression of several dark-induced genes (DINs) in *nbl1* and wild-type plants. We found that the expression of all six checked DINs in *nbl1* plants was significantly higher than that in wild-type plants (Fig. 2d-h). All results indicated that *nbl1* was more sensitive to dark-induced leaf senescence.

### The *nbl1* Mutant Was more Susceptible to *M. oryzae* and *Xoo*

**Fig. 3:**
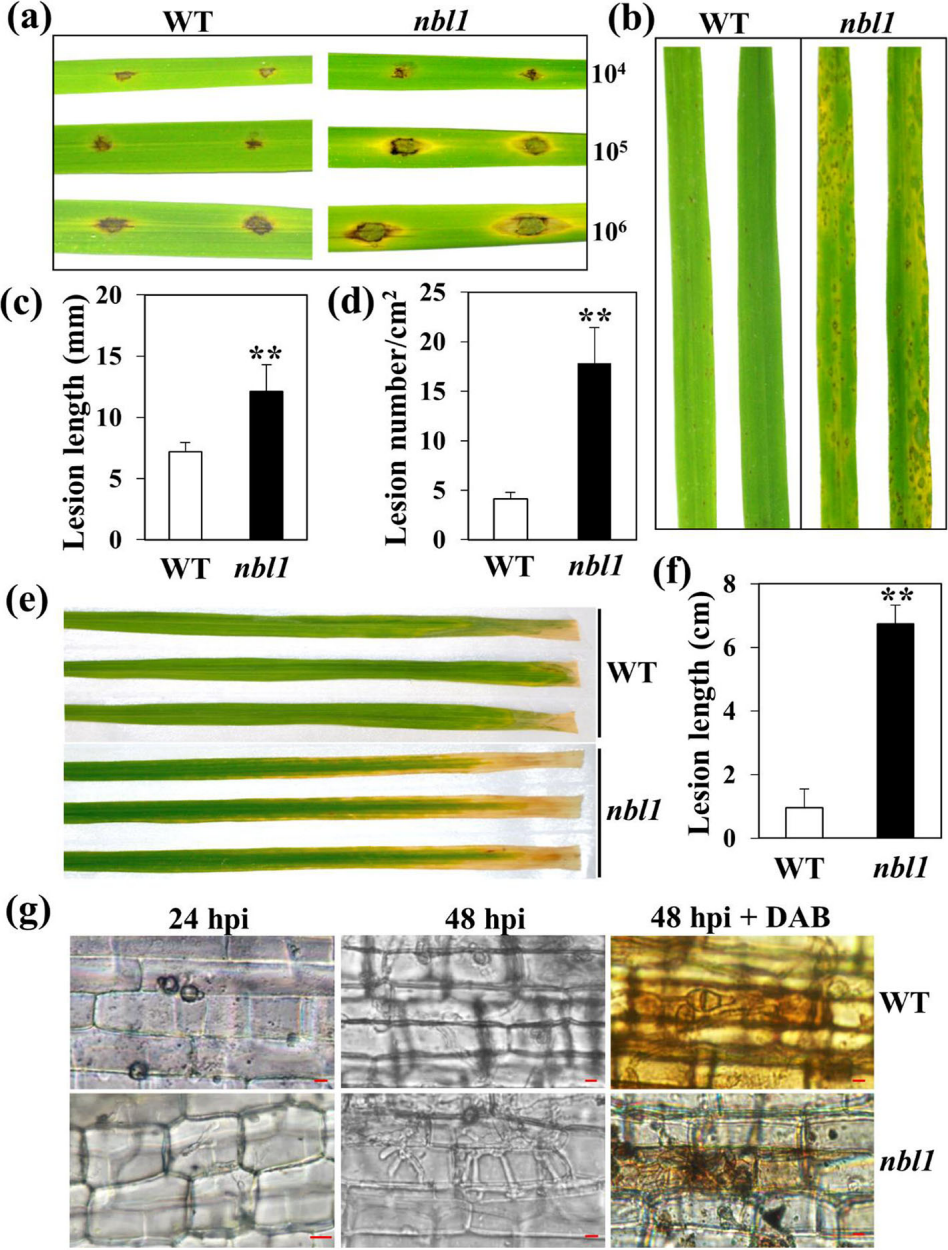
Determination the resistance of *nbl1* and WT plants to *M. oryzae* and *Xoo*. **a**-**d** Punch (**a**) and spray (**b**) inoculation of *nbl1* and WT plants with a weak virulent *M. oryzae* isolate 14–856-2. Pictures were photographed at 96 h post inoculation (hpi). 10^4^, 10^5^ and 10^6^ indicate three concentrations of 14–856-2, 1 × 10^4^ spores/mL, 1 × 10^5^ spores/mL and 1 × 10^6^ spores/mL, respectively. Lesion length (**c**) and lesion number (**d**) were measured at 96 hpi (1 × 10^6^ spores/mL). Values are means ± SD (n=3). **P < 0.01 (Student’s *t*-test). **e**-**f** Leaves of *nbl1* and WT plants were inoculated with *Xoo* isolate *PXO.99*. Picture of leaves (**e**) were photographed and lesion lengths (**f**) were measured at 14 days post inoculation (dpi). Values are means ± SD (*n*=10). **P < 0.01 (Student’s *t*-test). **g** Observation and record the infection process of 14–856-2 on the sheath cells of *nbl1* and WT plants by microscopy at 24 hpi and 48 hpi. DAB staining of infection sites was carried out at 48 hpi. Brown shading indicates accumulation of H_2_O_2_

Plant senescence is a form of programmed cell death (PCD), which involves plant immunity to pathogens (Daneva et al., 2016). To evaluate resistance to rice blast, one-month-old seedlings of the *nbl1* mutant and wild-type cv. Aichiasahi were inoculated with a weak but virulent isolate of *M. oryzae* (14–856-2). A spraying inoculation assay showed that the leaves of the *nbl1* mutant displayed severe lesions and chlorosis, while the wild-type exhibited near-immunity to 14–856-2 at 96 h post inoculation (Fig. 3b,d). Moreover, three concentrations of spore suspension of 14–856-2 were used to perform a punch inoculation assay on detached leaves of the *nbl1* mutant and wild-type. The lesions were larger on the leaves of *nbl1* than on the wild-type in each treatment (Fig. 3a,c). As the concentrations gradually increased, the lesions on the leaves of *nbl1* became more serious (Fig. 3a). We also inoculated three-month-old plants of *nbl1* and the wild-type with *X. oryzae* pv. *oryzae* strain *PXO99*. The lesions were much longer on *nbl1* leaves than on the wild-type at 14 days post inoculation (Fig. 3e,f).

To understand the differences in the *M. oryzae* infection process between *nbl1* and the wild type, isolate 14–856-2 of *M. oryzae* was used to carry out a sheath cell inoculation assay. Invasive hyphae formed on *nbl1* at 24 hpi (h post inoculation) and extended to the neighboring cells in large numbers at 48 hpi (Fig. 3g). In contrast, penetration pegs formed at 24 hpi and minimal invasive hyphae at 48 hpi on the wild type (Fig. 3g). DAB staining showed that ROS burst in the sheath cells of the wild type at 48 hpi, while little ROS was monitored in *nbl1* (Fig. 3g). These results indicate that pathogens induced ROS generation, an important signal transduction mechanism, which was interrupted in *nbl1,* reducing basal immunity.

To examine the mechanism of reduced disease resistance in *nbl1*, the expression of several defense-relative genes was detected by RT-qPCR. The expression of three *R* genes, *OsRPM1* (LOC_Os01g36640, rice homologs of Arabidopsis PM-associated protein RPM1; Cao et al., 2016), *OsRPP13* (LOC_Os10g36270; Ding et al., 2020), and *OsRP1L1* (LOC_Os05g30220; Wang et al., 2013), plays key roles in resistance to rice fungal and bacterial pathogens. The homologs of *Arabidopsis* genes RPS2 and RPP8, *OsRPS2* (LOC_Os09g10054) and *OsRPP8* (LOC_Os11g41540), were dramatically decreased in *nbl1* (Fig. S3). Another transcription factor, *OsWRKY45*, involved in the SA signaling pathway (LOC_Os05g25770, Shimono et al., 2012), was also significantly downregulated in *nbl1* (Fig. S3). These results suggest that the defense-related pathway was possibly weakened, thus contributing to reduced disease resistance in *nbl1*.

### The *nbl1* Mutant Showed Enhanced Salt Tolerance

**Fig. 4:**
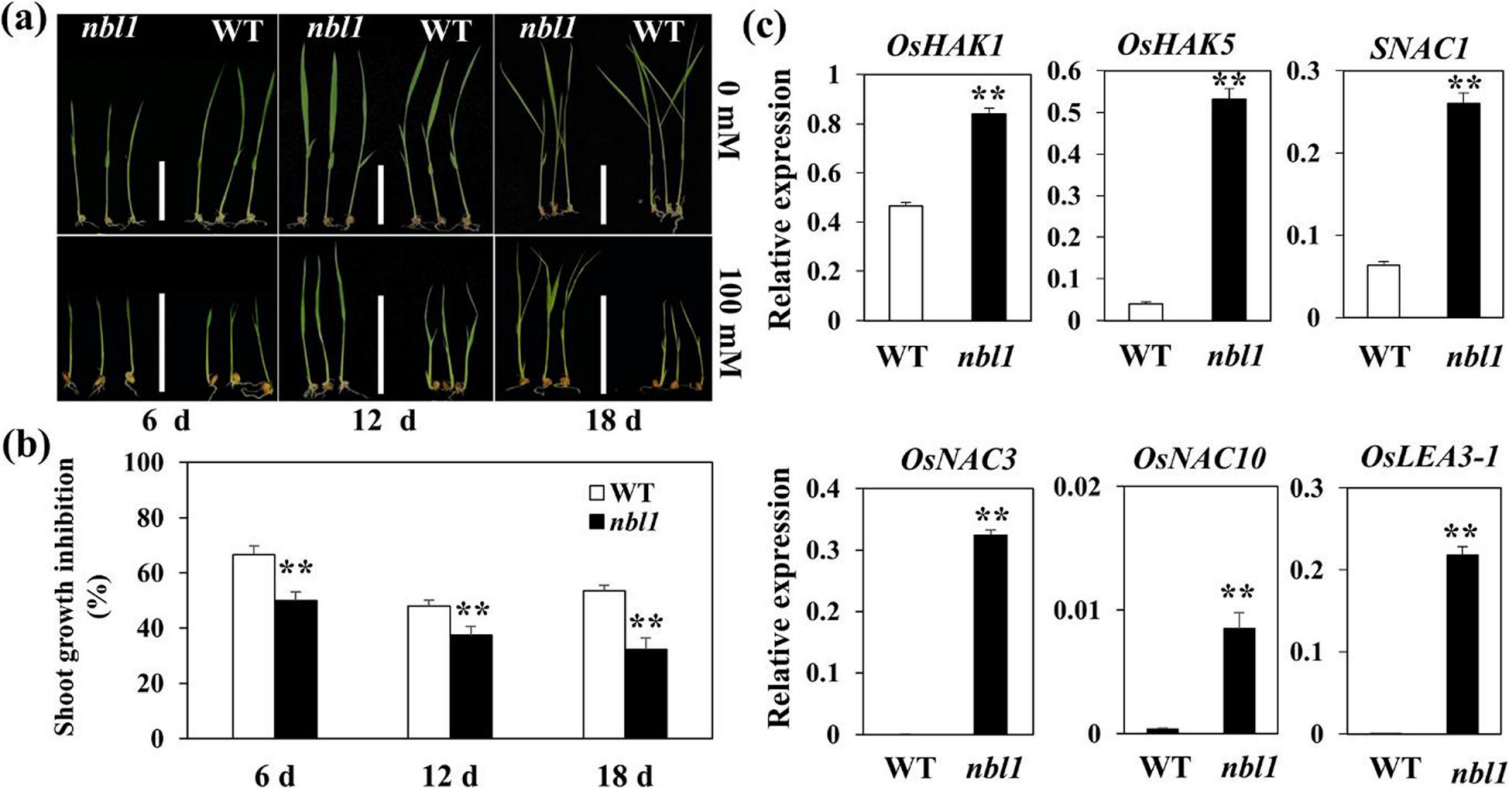
Phenotypic observation of *nbl1* and WT plants under salt treatment. **a** Observation and photograph the *nbl1* and WT plants under nutrition solution with 0 mM (control) and 100 mM NaCl at 6 d, 12 d and 18 d. Scale bar = 5 cm. **b** Measurement plant height and calculation the inhibitory ratio of seedlings height of *nbl1* and WT plants at the indicated day. Values are means ± SD (n=10). **P < 0.01 (Student’s *t*-test). **c** The expression of six salt-tolerance genes in the leaves of *nbl1* and WT plants were analyzed by RT-qPCR at seedling stage. Data were normalized to the expression of the *OsACTIN1* gene. Values are means ± SD (*n*=3). **P < 0.01 (Student’s *t*-test)

Environmental factors are external signals closely related to plant senescence (Schippers et al., 2015). Among them, salinity is one of the most important environmental stressors that promote leaf senescence. To test the *nbl1* response to environmental factors, we performed a salt tolerance analysis. Germinated seeds of *nbl1* and the wild type were cultured in an incubator with nutrient solution containing 0 (control) and 100 mM NaCl for 6, 12, and 18 d. The plant height of *nbl1* was shorter than that of the wild type in all three assessment times in control conditions (0 mM; Fig. 4a,b). Conversely, in the nutrient solution containing 100 mM NaCl, the plant height of *nbl1* and the wild-type were similar at 6 d; at 12 d and 18 d, the plant height of *nbl1* was much higher than that of the wild type. The wild type displayed a more severe premature senescence phenotype at 18 d after treatment (Fig. 4a,b).

To further understand the mechanism of salt tolerance in *nbl1*, we analyzed the expression of several salt-tolerance genes by RT-qPCR. *HAK1* and *HAK5*, which are K^+^ transporters that maintain the balance of sodium and potassium under salt stress and ensure normal plant growth (Horie et al., 2009), were significantly up-regulated in *nbl1* (Fig. 4c). We also found that the expression of three NAC transcription factors, *OsSNAC1*, *OsNAC3,* and *OsSNAC10*, which are involved in drought and high salinity resistance (Hu et al., 2006; Jeong et al., 2010; Liu et al., 2011), were also obviously increased in *nbl1* (Fig. 4c). The expression of a *LEA* (Late-Embryogenesis Abundant protein) gene, which could improve rice tolerance to drought in field conditions (Xiao et al., 2007), was about 50 times higher in *nbl1* than in the wild type (Fig. 4c). These results suggest that several salt tolerance pathways were activated, causing enhanced resistance to salt and salt-induced senescence in *nbl1.*

### The *nbl1* Mutant Was more Sensitive to Exogenous Salicylic Acid (SA)

**Fig. 5:**
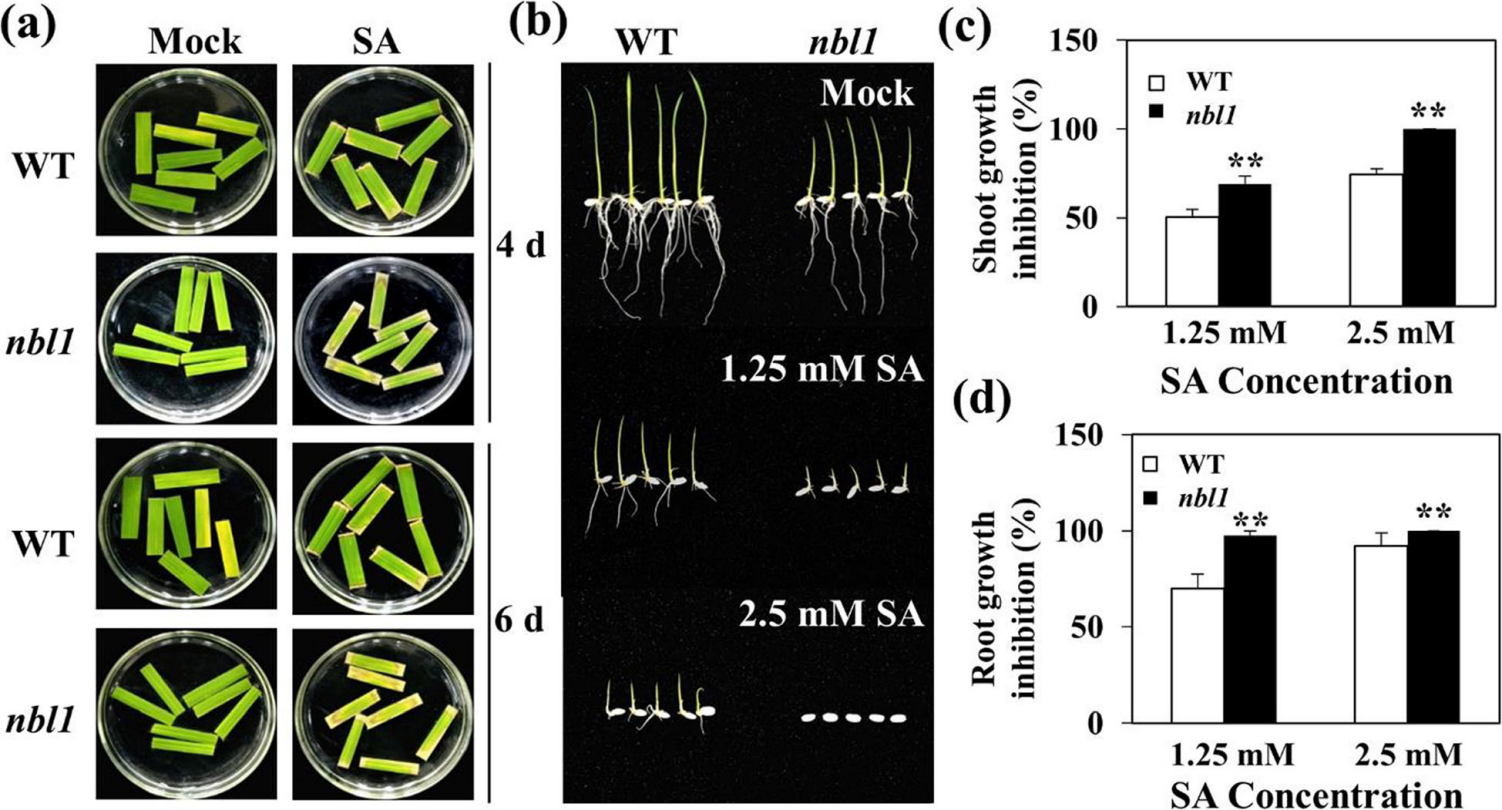
Responses to exogenous SA. **a** Detached leaves of six-weeks-old seedlings of *nbl1* and wild-type plants were immersed in buffer solution (3 mM MES, pH 5.8) with 0 μM (Mock) or 100 μM SA for 6 days at room temperature (24 h light). Photographed at 4 days and 6 days. **b**-**d** Phenotypes of *nbl1* and wild-type seedlings grown on the 1/2 MS containing the indicated concentrations of SA for 6 days B). Measurement the shoot and root length of *nbl1* and WT plants, then calculation the inhibitory ratio of shoots length (**c**) and roots length (**d**), respectively

Phytohormones are important internal signals that regulate plant senescence (Schippers et al., 2015). To study the influence of exogenous phytohormones on the function of senescence in *nbl1*, detached leaves of six-week-old *nbl1* and wild-type seedlings were soaked in control, ABA (50 μM), ACC (10 mM), MeJA (100 μM), and SA (100 μM) solutions. All treatments were kept under 24 h light, and the leaf fragments were photographed at 0, 2, 4, and 6 d. Among the four phytohormones, the leaf senescence induced by ABA (50 μM), ACC (10 mM), and MeJA (100 μM) was not significantly different between *nbl1* and the wild type (Fig. S4). Nevertheless, SA (100 μM) accelerated leaf senescence in *nbl1*, which first appeared at 4 d and was more obvious at 6 d than that in the wild type (Fig. 5a; Fig. S4). To further understand the effect of SA, we germinated nbl1 and wild-type seeds in 1/2 MS culture medium containing 0 (control), 1.25, and 2.5 mM SA, and measured the shoot and root length after 6 d in the greenhouse (28 °C, photoperiod, 16 h:8 h, light:dark). Compared with the wild type, the shoot and root length of *nbl1* was much more inhibited in media containing 1.25 mM SA, and *nbl1* did not survive in the medium with 2.5 mM SA (Fig. 5b,c). These results indicated that *nbl1* was more sensitive to SA.

### Cloning of *OsNBL1*

The *nbl1* mutant was identified from a T-DNA insertion pool. Genetic analysis of the heterologous *nbl1* offspring indicated that the leaf senescence phenotype was co-segregated with the T-DNA insertion at a ratio of ∼1:3. We also crossed the homozygous *nbl1* with the wild-type cultivar ‘Nipponbare’. The mutant phenotype and wild-type phenotype segregated at a ratio of 1:3 in the F2 population of *nbl1*/Nipponbare (Table S1). These results suggested that *nbl1* was a recessive mutation caused by T-DNA insertion. The flanking sequence of T-DNA was obtained by TAIL-PCR. Sequence analysis showed that the T-DNA was inserted in the coding region of *LOC_Os10g33855*. The T-DNA insertion site was 4-bp upstream of the stop codon site of *LOC_Os10g33855*, as confirmed by site-specific PCR (Fig. S5a,b). RT-qPCR analysis showed that the expression of *LOC_Os10g33855* in *nbl1* seedlings was significantly lower than that of the wild type (Fig. S5c), suggesting that *LOC_Os10g33855*, thereafter renamed *OsNBL1,* was the only gene within the vicinity of the T-DNA insertion.

To verify the causality of *OsNBL1* for the *nbl1* phenotype, we generated a construct, *p35S:NBL1-GFP*, in which the full-length CDS of *LOC_Os10g33855* was fused to eGFP under the control of the CaMV 35S promoter, which was transformed into *nbl1*. Transgenic plants (15) were screened out by amplifying the eGFP gene as a selective marker. All transgenic plants (represent Com-1, Com-2, and Com-3) rescued the senescence phenotype of *nbl1* (Fig. S6). This result demonstrated that the *nbl1* phenotype resulted from the disruption of *OsNBL1*.

### *OsNBL1* Encoded a Conserved Protein in both Monocot and Dicot Plants

According to annotation by The Rice Genome Annotation Project (http://rice.plantbiology.msu.edu/), two transcripts of *OsNBL1* were predicted, one of which encoded a long-chain protein with 141 amino acids, while the other encoded a short-chain protein with 94 amino acids. There was only one transcript which encodes a short-chain protein with 94 amino acids predicted by The Rice Annotation Project (https://rapdb.dna.affrc.go.jp/). Moreover, using the flanking sequence of *OsNBL1* as a query, only one cDNA sequence with accession No. AK062836 that encoded the short-chain protein was searched out. Furthermore, we cloned the full-length CDS of *OsNBL1* from rice cv. Aichiasahi, and sequencing data showed that it is 285 bp in length and encodes a protein with 94 amino acids (Fig. S5f). Based on BLASTP search using the OsNBL1 sequence as the query, a total of 15 OsNBL1 homologs were picked up from monocot and dicot plant species. Sequence alignments showed that all OsNBL1 homologs had a highly conserved transmembrane domain at the C terminus (Fig. S5d). Phylogenetic analysis showed that OsNBL1 shared higher amino acid sequence identity with proteins in monocot species, such as *Sorghum bicolor* (XP_002467074.2, 65.88%), *Setaria italica* (XP_004982857.1, 64.77%), *Zea mays* (XP_008644776.1, 63.53%), and *Brachypodium distachyon* (XP_003574064.1, 63.24%) (Fig. S5e). The high sequence similarity of OsNBL1 homologs in both monocots and dicots suggests that they may perform similar functions, although none of them is functionally characterized.

### Overexpression of *OsNBL1* Led to Delayed Leaf Senescence but Did Not Affect Disease Resistance

**Fig. 6:**
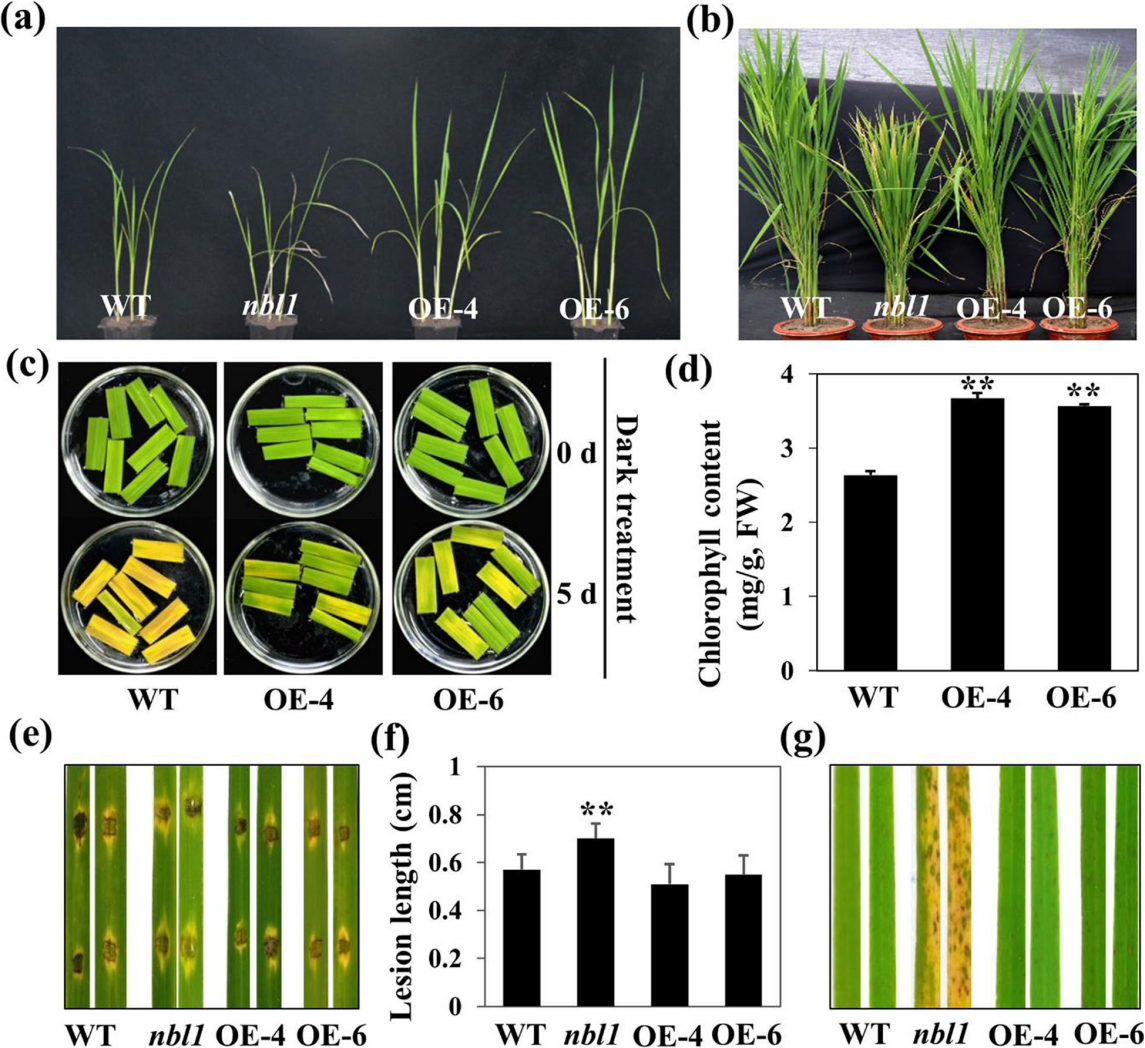
Evaluation to dark-induced senescence and blast resistance of *OsNBL1* OE plants. **a**-**b** The phenotype of WT, *nbl1*, OE-4 and OE-6 plants at seedling stage (**a**) and heading stage (**b**). **c** Detached leaves of WT, OE-4 and OE-6 plants grown for six-weeks were immersed in buffer solution (3 mM MES, pH 5.8) at 28 °C for 5 days with complete darkness. **d** Total chlorophyll of the flag leaves of WT, OE-4 and OE-6 plants were measured at heading stage. Data are means ± SD (n=3). **P < 0.01 (Student’s *t*-test). **e**-**g** Punch (**e**) and spray (**g**) inoculation of WT, *nbl1*, OE-4 and OE-6 plants with a virulent *M. oryzae* isolate H535. Photograph and measurement of lesion length (**f**) was at 96 hpi

To further determine the function of *OsNBL1* in regulating senescence and defense, full-length CDS of *OsNBL1* driven by a ubiquitin promoter was introduced into the wild type, and 20 transgenic plants were obtained and confirmed by RT-qPCR (Fig. S7). Overexpression plants, OE-4 and OE-6, exhibited higher plant height than the wild type at the seedling stage (Fig. 6a); however, it was not significantly different at the heading stage (Fig. 6b). Moreover, OE-4 and OE-6 plants displayed delayed dark-induced leaf senescence and increased chlorophyll content compared with wild-type plants (Fig. 6c,d). In addition, *nbl1*, the wild type, and overexpression plants of *OsNBL1*, OE-4 and OE-6, were challenged with isolate H535 of *M. oryzae*. Both spraying and punch inoculation assays indicated that the lesions on *nbl1* leaves were more numerous and larger than those of wild-type, OE-4, and OE-6 plants. However, the lesion number and size were not significantly different between the wild type and the two *OsNBL1* overexpression lines (Fig. 6e-g). These results indicated that overexpression of *OsNBL1* could improve the chlorophyll content and delay dark-induced leaf senescence but not affect disease resistance in rice.

### OsNBL1 Is a Multi-Organelle Localized Protein

**Fig. 7:**
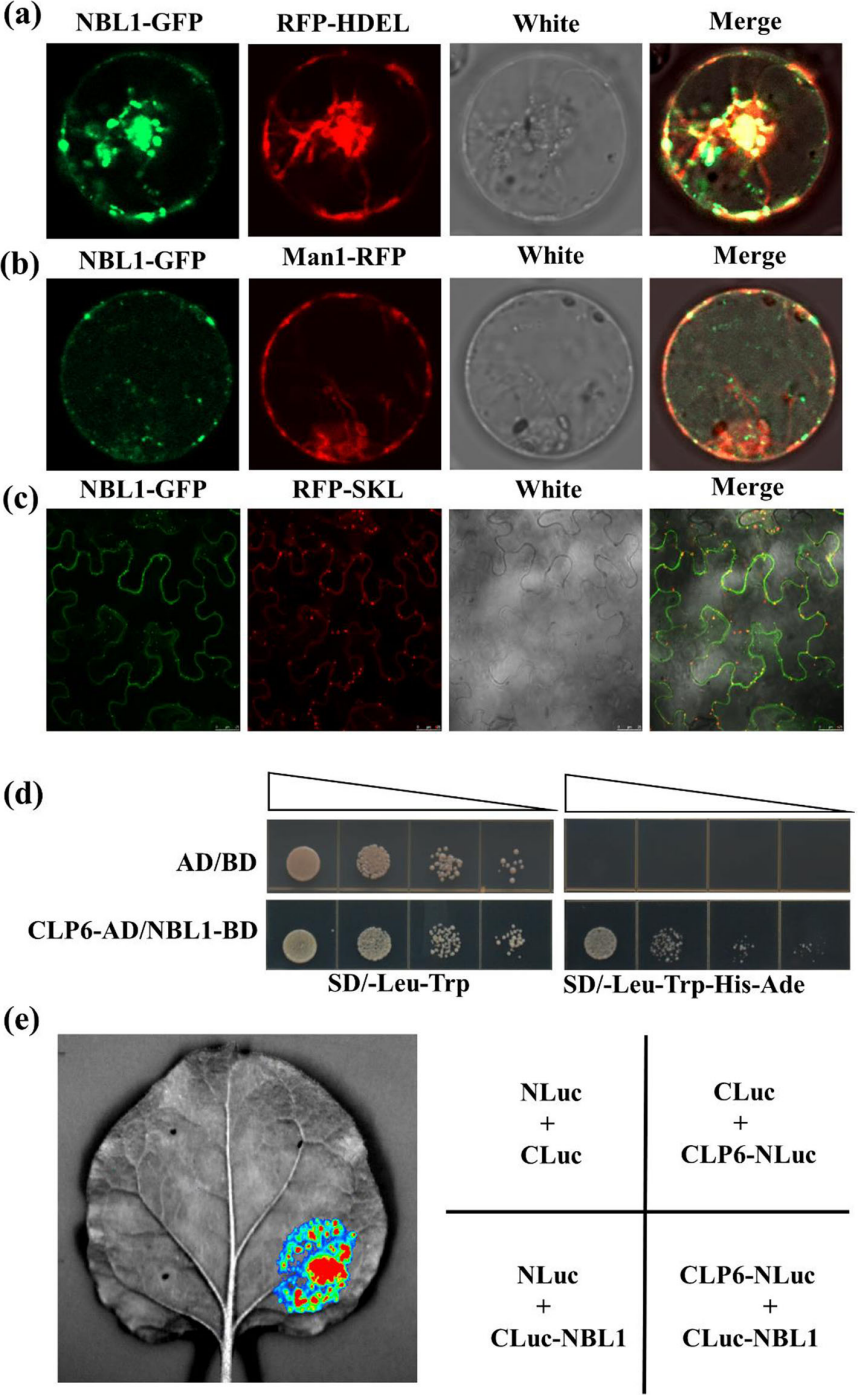
OsNBL1 is a multi-localized protein and interacts with OsClpP6. **a**-**c** OsNBL1-GFP was transiently co-expressed with RFP-HDEL (**a**) or Man1-RFP (**b**) in rice protoplasts. The (**c**) shows the co-expression of OsNBL1-GFP and RFP-SKL in the *N. benthamiana* leaves by agrobacterium-mediated transformation. RFP-HDEL, Man1-RFP and RFP-SKL indicate the marker protein of endoplasmic reticulum (ER), Golgi and peroxisome, respectively. **d** Y2H shows that OsNBL1 interacts with OsClpP6. **e** The LCI assay indicates the OsNBL1-OsClpP6 interaction in *N. benthamiana* leaf cells. Luminescence was detected with a cooled CCD imaging system at 48 h after co-infiltration of each group of constructs


To examine the subcellular localization of the OsNBL1 protein, the *p35S: NBL1-GFP* construct was transiently transformed into rice protoplasts and *Nicotiana benthamiana* epidermal cells. The NBL1-GFP signal was distributed in the cell membrane and cytoplasm, in which it formed a dynamically moving punctate structure, while GFP alone coated the cell (Fig. S8 and Movie S1). Given that the NBL1-GFP spot was moving in the cytoplasm, we speculated that OsNBL1 may be involved in endomembrane transport systems. To test this possibility, we used NBL1-GFP with RFP-HDEL (ER marker) and Man1-RFP (Golgi marker) to perform a co-expression transient transformation in rice protoplasts. The punctate compartment signals of NBL1-GFP were obviously overlapped with ER and Golgi trackers (Fig. 7a,b). Moreover, NBL1-GFP was transiently co-expressed with RFP-SKL (peroxisome marker) in *Nicotiana benthamiana* epidermal cells. The punctate compartment signals of NBL1-GFP were also partially merged with the peroxisome tracker (Fig. 7c). These results indicated that OsNBL1 is a multi-organelle localized protein and may be involved in endomembrane systems transport.

**Additional file 1: Movie S1.** Time-Lapse observation of OsNBL1-GFP expressing in *N. benthamiana* leaf epidermal cells by microscopy.

### OsNBL1 Interacts with OsClpP6

To understand the biochemical function of OsNBL1, we performed Y2H screening to search interaction proteins using OsNBL1-BD as bait, and four candidate OsNBL1 Interaction Proteins (NIPs) were screened out (Fig. S9). Among the NIPs, one protein, OsClpP6, was previously reported to be involved in leaf senescence in rice (Dong et al., 2013). The interaction between OsNBL1 and OsClpP6 was re-confirmed by a point-to-point Y2H assay (Fig. 7d). Furthermore, we conducted a firefly luciferase complementation imaging (LCI) system to validate the interaction of OsNBL1 and OsClpP6 in *N. benthamiana* leaves. Using a low-light imaging system, the fluorescence signals were detected when CLuc-OsNBL1 and OsClpP6-NLuc were co-expressed in *N. benthamiana* leaves (Fig. 7e). These results indicated that OsNBL1 interacted with OsClpP6.

## Discussion

### OsNBL1 Is a Novel Regulator of Leaf Senescence, Salt Tolerance, and Disease Resistance in Rice

Leaf senescence is an inevitable stage of annual crop plants in a growing season, yet premature senescence leads to reduced crop yield. It is a complex process that is not only controlled by developmental age but also regulated by a variety of internal signals and external factors, including phytohormones, dark, high salinity, and pathogen attack, among others (Lim et al., 2007; Jibran et al., 2013; Schippers et al., 2015). These factors stimulate plants to make a response, which is a multiple hierarchical signaling pathway regulated by complex gene networks (Zhang et al., 2020). In the past several decades, a growing number of leaf senescence-associated genes (e.g., transcription factors, 3-oxoacyl-ACP reductase, MAPKs, acyl hydrolase, Histone deacetylases, and H3K4 demethylase, etc.), involving a diversity of biological processes, have been cloned and identified to clarify the mechanism of plant leaf senescence (Niu et al., 2020; Zhou et al., 2020; Guo et al., 2017; Zhang et al., 2017; He & Gan, 2002; Zhou et al, 2009; Zhao et al., 2020; Liu et al., 2019). Although many such genes have been characterized to understand the process of leaf senescence, there still remain many unknowns, and many more genes need to be identified. In this study, we isolated and characterized a recessive premature senescence mutant *nbl1*, which displayed growth retardation, yellowish leaf tips on old leaves of the lower part of the plant at the seedling stage, and accelerated senescence of all leaves at tillering and heading stages (Fig. 1a-c).

In this study, we demonstrated that the disruption of *OsNBL1* was responsible for *nbl1* phenotypes. *OsNBL1* encoded an unknown function protein with a transmembrane domain, and phylogenetic analysis suggested that the homologous relationship of OsNBL1 was more similar to monocot plants (Fig. S5e). As far as we know, the functions of OsNBL1 and its orthologues in plants have not been reported. The degradation of chlorophyll is a very important index in the process of plant leaf senescence (Lim et al., 2007). The *nbl1* plants displayed lower chlorophyll content and a higher transcription level of several chlorophyll degradation-related marker genes (Fig. 1b-i). Moreover, both whole seedlings and detached leaves of *nbl1* showed accelerated senescence when under darkness for 3 or 4 days (Fig. 2a,b). RT-qPCR analysis indicated that mRNAs of *DINs* were excessively accumulated in *nbl1* leaves (Fig. 2c). In contrast, the overexpression lines of *OsNBL1* plants showed higher chlorophyll content and delayed dark-induced leaf senescence (Fig. 6c,d). Our results suggested that loss of function in *OsNBL1* leads to constitutive activation of the expression of several senescence-associated genes, causing chlorophyll degradation and premature senescence in *nbl1*. Thus, OsNBL1 is a novel negative regulator of leaf senescence in rice.

Although *nbl1* displayed a premature senescence phenotype, it was found to have enhanced tolerance to high salt. The salt stress assay showed that the growth of *nbl1* was less inhibited in nutrient solution with 100 mM NaCl (Fig. 4a,b). Several salt-related genes, namely a K^+^ transporter, NAC transcription factor, and Late-Embryogenesis Abundant protein, were up-regulated in *nbl1* (Fig. 4c). It has been known that plants exposed to high salt stress would produce ROS, which play a dual function in the salinity response (Jiang et al., 2012). ROS are important molecules for active cellular signal transduction but are also toxin oxides to cells, leading to reduced salt tolerance. For example, the *ein3* mutant showed excessive ROS accumulation when treated with NaCl, while fewer ROS were generated in overexpression plants of *EIN3*, suggesting that *EIN3* enhanced salt tolerance by deterring ROS accumulation (Peng et al., 2014). In addition, transgenic plants with overexpressing enzymes, like glutathione peroxidase (GPX), superoxide dismutase (SOD), and ascorbate peroxidase (APX), improved the capability for scavenging ROS and exhibited enhanced salt tolerance (Peng et al., 2014). In this study, fewer ROS were found in the leaves of *nbl1* than in those of the wild type (Fig. S2). According to these findings, we speculated that OsNBL1 may inhibit the process of scavenging ROS, resulting in reduced salt tolerance in rice.

Leaf senescence is a pattern of programmed cell death (PCD), which plays a significant role in plant immunity (Daneva et al., 2016), and several studies have indicated that many premature senescence mutants show enhanced resistance to pathogens (Zhang et al., 2020). However, *nbl1* was more susceptible to *M. oryzae* and *X. oryzae*, with defense-related genes down-regulated (Fig. 3a-f). The overexpression plants of *OsNBL1*, OE-4 and OE-6, exhibited similar resistance to *M. oryzae* to that of wild type plants (Fig. 6e-g). ROS levels were rapidly elevated during ETI and PTI, involving the activation of Ca^2+^ influx in the cells, MAPK cascades, and transcription factors, which contributed to disease resistance (Wrzaczek et al., 2013). DAB staining indicated that pathogen-induced ROS generation was blocked in *nbl1*, and investigation of the infection process displayed that *M. oryzae* strain 14–856-2 invaded and expanded rapidly on *nbl1* leaves (Fig. 3g). A lower content of ROS was identified in *nbl1* leaves under normal growth conditions, suggesting that OsNBL1 is necessary for ROS accumulation for active plant immunity in rice.

SA has been reported as a key phytohormone that participates in leaf senescence, salt tolerance, and plant immunity (Zhang et al., 2020). The *nbl1* plants exhibited accelerated leaf senescence and remarkable inhibition of seedling growth compared to the wild type when treated with exogenous SA (Fig. 5), indicating that loss of function in OsNBL1 might reduce the metabolic capacity of SA or might activate signal transduction of SA pathways in rice. Previous reports revealed that plant H_2_O_2_ scavenging enzymes were inactivated by SA, leading to the accumulation of H_2_O_2_; conversely, SA content is increased by exogenous application of H_2_O_2_, promoting SA biosynthesis (Guo et al., 2017). ROS and SA also synergistically regulate plant responses to senescence and environmental stress (Zhang et al., 2020). In this study, the SA pathway was activated, while the ROS signaling pathway was interrupted in *nbl1,* causing a senescence phenotype. Notably, activation of the SA pathway promoted leaf senescence and enhanced salt tolerance but impaired plant immunity in *nbl1*. These results suggest that OsNBL1 plays a negative role in SA induced leaf senescence but acts as a positive factor in SA-dependent plant defense. Taken together, NBL1 is a novel factor participating in ROS generation and SA signal pathway, ant that is involved in balancing SA and ROS pathways, controlling leaf senescence, salt tolerance, and disease resistance in rice.

### OsNBL1 Is a Multi-Localized Protein, Suggesting that it Is Involved in Versatile Cell Processes

The endomembrane system is a complex network for transporting and exchanging proteins, lipids, and other materials, playing an important function in plant development and responding to various environmental stimuli (Morita & Shimada, 2014). In this study, *OsNBL1* encoded an small molecular protein of unknown function with 94 amino acids, containing a putative transmembrane domain, indicating that OsNBL1 may work on the membrane system. Subcellular localization results showed that OsNBL1 was located primarily in the ER but also in the Golgi, formed a punctate structure, and dynamically moved in the cytoplasm (Fig. 7a,b; Movie S1), suggesting that OsNBL1 may be involved in endomembrane system trafficking. Furthermore, OsNBL1 was localized on peroxisomes (Fig. 7c). Plant peroxisomes are probably the major sites for production of intracellular ROS, which is a dual factor regulating senescence and environmental stress (Del Río & López-Huertas, 2016). Thus, OsNBL1 is a multi-localized protein, and we speculated that it is involved in versatile cell processes.

In high plants, Clps have been proven to act in essential roles for controlling the quality and quantity of chloroplast proteins involved in chloroplast biogenesis and function (Adam et al., 2006). One subcomplex of Clps, the P ring, which consists of four subunit proteins, ClpP3, ClpP4, ClpP5, and ClpP6, with a ratio in 1:2:3:1 (Olinares et al., 2011a), is synthesized on the endoplasmic reticulum in the cytosol (Sakamoto, 2006; Jarvis & López-Juez, 2014). In Arabidopsis, the loss of function of any subunit on the P ring leads to embryo or seedling death, whereas partial down-regulation of the subunit genes, such as *CLPP4* and *CLPP6*, results in varying degrees of leaf chlorotic phenotypes in all growth stages (Kuroda & Maliga, 2003; Peltier et al., 2004; Sjögren et al., 2006; Zheng et al., 2006; Kim et al., 2009; Olinares et al., 2011b). Moreover, each subunit has a unique function in the Clp complex (Halperin et al., 2001; Andersson et al., 2009; Kim et al., 2013). Notably, the correct ratio of each subunit plays an important role in the overall structure of the Clp complex, and disruption of any subunit might break the function of the Clp protease complex (Shen et al., 2007). The protein levels of ClpP1, ClpP5, and ClpP6 accumulate in *clpP3* mutants (Kim et al., 2013), and in the antisense plants of *ClpP6*, ClpP4 and ClpP5 subunits are increased, while ClpP3 is reduced (Sjögren et al., 2006; Kim et al., 2013). Recently, a study showed that the E3 ligase AtCHIP can interact with ClpP3 and ClpP5 in yeast and ubiquitylate ClpP3 and ClpP5 in vitro, controlling the homeostasis of Clp subunits (Wei et al., 2015). In rice, *loss of function in ClpP6 retarded plant growth and caused chlorosis in leaves (*Dong et al., 2013*). However, the* regulation of ClpP6 in rice remains unclear.

In this study, we used OsNBL1 as a bait to screen the Y2H cDNA library of rice and identified an interactor protein OsClpP6 (Fig. 7dc). The interaction between OsNBL1 and OsClpP6 was further confirmed by a LCI assay (Fig. 7e). In consideration of the similar phenotype of *nbl1* and *clpC6* mutants, we speculated that OsNBL1 might affect the function of OsClpP6 to regulate plant growth and development. However, how OsNBL1 modulates OsClpP6 is still unknown. For example, it is unclear if the activity and/or the protein level of OsClpP6 is affected by OsNBL1. It will be interesting to clarify the details of how OsNBL1 regulation affects OsClpP6 in further studies, which may provide new insights in leaf senescence and plants’ response to biotic and abiotic stresses.

## Conclusions

Here, it is showed that OsNBL1, a multi-organelle localized protein which interacts with a plastidic caseinolytic protease OsClpP6, is essential for leaf senescence and environmental stresses. The null mutant *osnbl1* exhibited accelerated leaf senescence and enhanced salt-tolerance but reduced disease resistance in rice, thus providing a new insight for revealing the mechanism of leaf senescence and response to environmental stresses, and a way to improve agricultural traits of crop plants.

## Methods

### Plant Materials and Growth Conditions

The *nbl1* mutant was isolated from a T-DNA insertion population of rice cv. ‘Aichiasahi’. All plants, including *nbl1*, the wild type, OE lines, and complementation plants, were cultivated in a paddy field under a normal growing season in the experimental field of China Agricultural University (Beijing) or grown in the greenhouse (28 °C, photoperiod, 16 h: 8 h, light: dark). Agronomic traits were measured during the growing season.

### Dark-Induced Senescence Treatment

Flag leaves of *nbl1*, OE lines, and wild-type plants grown in field conditions were cut into about 3 cm fragments at the heading stage. Fragments were soaked in petri dishes with 15 ml MES buffer (10 mM, pH=5.7). Three-week-old rice seedlings of *nbl1* and wild-type plants grown in the greenhouse were moved into black boxes. After 4 days or 3 days of full darkness at 28 °C, leaf fragments and plants were assessed and photographed. Each treatment was repeated three times.

### Measurement of Chlorophyll Content

For chlorophyll extraction, 0.2 g fresh flag leaves of *nbl1*, OE lines, and wild-type plants were ground sufficiently with a mortar and pestle with about 20 mL of 90% ethanol, CaCO_3_, and an appropriate amount of arenaceous quartz. Then, the mixture was filtered to obtain the supernatant, which was transferred into a clear tube and a final volume of 50 mL was made with 90% ethanol. The absorbance of chlorophyll solution at 652 nm was measured by a spectrophotometer (UV-2450, Shimadzu). All experiments were carried out three times. The chlorophyll content was calculated according to a previous report (Arnon, 1949).

### Pathogen Inoculation and Infection Process of *M. oryzae* Examination

The inoculation method of *M. oryzae* was based on a previous report (Fang et al., 2018). In brief, isolates of *M. oryzae* were cultured on oatmeal-tomato agar plates for 7 days and used to induce conidia production. Conidial suspensions were adjusted to 1 × 10^4^, 1 × 10^5^, and 1 × 10^6^ spores/mL in 0.025% Tween 20 solution. Detached leaves of fifth leaf-stage rice seedlings were scratched and inoculated with a drop of 10 μL of different concentrations of the conidial suspension. Moreover, the conidial suspension (1 × 10^6^ spores/mL) was spray-inoculated on intact leaves. Both the scratched and intact inoculated leaves were transferred to a chamber for 24 h in the dark at 28 °C and 100% humidity, then converted to light conditions (photoperiod, 24 h light). The lesion length was measured and photographed at 96 h. For investigation of the infection process of *M. oryzae*, the second leaf sheaths (from top to bottom) were cut from four-week-old seedlings and injected with 1 × 10^5^ spores/mL conidial suspensions. All images of *nbl1* and wild-type plants with inoculation at 24 and 48 h were recorded by micrography (ECLIPASE 90i, Nikon). Details of operation were described in a previous report (Kankanala et al., 2007).

The leaf-clipping method was used to inoculate *nbl1* and wild-type plants with the *Xoo*. *oryzae* pv *oryzae* strain *PXO99*. Bacteria were cultured overnight and collected, then resuspended with deionized water and adjusted to a concentration of OD_600_=0.8 for inoculation. Flag leaves of 60-day-old plants grown in a greenhouse were inoculated by removing the leaf tips (about 3 cm) with scissors dipped in the bacterial suspension. Infected plants were grown in a glasshouse, and the lesion length was measured at 14 days post inoculation (dpi). All experiments were repeated three times.

### DAB and NBT Staining

DAB and NBT staining were carried out as previously described with a simple modification (Qiao et al., 2010). Briefly, leaves of *nbl1* and wild-type were immersed in DAB (3,3′-diaminobenzidine) solution (1 mg/mL DAB, 10 mM Na_2_HPO_4_, pH 3.8) or NBT (nitro blue tetrazolium) solution (0.5 mg/mL NBT, 10 mM K_2_HPO_4_, pH 7.8) in the dark for 16 h at room temperature. Subsequently, the stained leaves were transferred into 90% ethanol to be decolorized. The cleaned leaves were photographed. In addition, DAB staining was performed on leaf sheaths that were inoculated with *M. oryzae*, and then examined by micrography. Each treatment was repeated three times.

### NaCl Tolerance Test

Seeds of *nbl1* and the wild-type were treated with sodium hypochlorite solution (1%) to accelerate germination. Germinated Seeds were planted in Yoshida solution containing 0 or 100 mM NaCl in the greenhouse (28 °C, photoperiod, 16 h: 8 h, light: dark), and the nutrient solution was changed once a day. Seedlings were photographed and the plant height was measured after 6, 12, and 18 d. Each treatment was repeated three times. Percentage of shoot growth inhibition was calculated as this formula: [L_(Control)_-L_(NaCl)_]/ L_(Control)_. L_(Control)_ is the shoot length of seedling in 0 mM NaCl, and L_(NaCl)_ indicates the shoot length of seedling in 100 mM NaCl.

### Exogenous Phytohormone Treatment

Detached leaves of six-week-old seedlings of *nbl1* and wild-type plants were used for evaluating the senescence phenotype to four senescence-promoting phytohormones, ABA, ACC, SA, and MeJA. The leaf fragments were immersed in buffer solution (3 mM MES, pH 5.8) with ABA (50 μM), ACC (10 mM), MeJA (100 μM), and SA (100 μM) under 24 h light for 6 d at room temperature. To test the effect of SA on germination and growth, seeds of *nbl1* and the wild-type were surface sterilized [in 70% ethanol for 10 min, then in sodium hypochlorite solution (4%) for 20 min, and washed with sterile water several times] and cultured in 1/2 Murashige & Skoog (MS) medium containing 0, 1.25, or 2.5 mM SA for 6 days in the greenhouse (28 °C, Photoperiod, 16 h: 8 h, light: dark). The leaf senescence phenotype and seedlings were photographed, and the shoot and root length were measured. Each treatment was repeated three times. Percentage of shoot/root growth inhibition was calculated as this formula: [L_(Control)_-L_(SA)_]/ L_(Control)_. L_(Control)_ is the shoot/root length of seedling in 0 mM SA, and L_(SA)_ indicates the shoot/root length of seedling in 1.25/2.5 mM SA.

### Genetic Analysis and Gene Mapping

For genetic analysis, T1 plants of *nbl1* and F2 plants crossing with *nbl1*and cv. ‘Nipponbare’ were grown in a paddy field, and the separation ratio of wild-type to mutant plants was counted. For mapping *OsNBL1*, TAIL-PCR was used as previously reported (Liu et al., 1995). In brief, the flanking sequence was amplified by PCR using three specific primers of the right border of T-DNA and arbitrary degenerate (AD1) primers. PCR products were sequenced, and BLAST analysis was done with a rice database (http://rice.plantbiology.msu.edu/) to determine the T-DNA insertion site. Primers P1, P2, and P3, which were specific primers designed from the genome and T-DNA, were used to confirm the insertion site of T-DNA. All primers for gene mapping are listed in Table S1.

### Phylogenetic Analysis of OsNBL1 Proteins

The conserved domains of OsNBL1 were predicted on the SMART website (http://smart.embl-heidelberg.de/smart/set_mode.cgi?NORMAL=1), and the homologous sequences of OsNBL1 were download from NCBI (http://blast.ncbi.nlm.nih.gov/). Multiple sequence alignments were performed, and the phylogenetic tree was built using MEGA X software with the Neighbor-joining method. The bootstrap values were calculated from 1000 replicates.

### Plasmid Construction and Rice Transformation

For generating complementary plants, full-length CDS sequences of *OsNBL1* were amplified by PCR using the primers OsNBL1-GFP-F/R, and the *OsNBL1* fragments were then cloned into a pCG1301 vector to generate the *p*3*5S:OsNBL1-GFP* construct, which was introduced into the *nbl1* background. Moreover, primers OsNBL1-OE-F/R were used to amplify the *OsNBL1* sequence, which was inserted into the pCAMBIA1301-UBI vector for generating the *pUBI:OsNBL1* construct*.* The *pUBI:OsNBL1* vector was transformed into the wild type to obtain overexpression plants. Rice transformation was performed using the *Agrobacterium tumefaciens*-mediated method (Nishimura et al., 2006). The sequences of primers used in this study are listed in Table S1.

### Real-Time Quantitative PCR (RT-qPCR) Analysis

RT-qPCR was performed to analyze the transcription levels of *OsNBL1*, dark-induced genes (*DINs*), senescence-associate genes (*SAGs*), and salt-tolerance genes in *nbl1* and wild-type plants. Total RNA was extracted from one-month-old seedling leaves using TRIzol reagent (Invitrogen), and the cDNA synthesis was conducted using HiScript III RT SuperMix for qPCR kit (Vazyme). RT-qPCR was performed using SYBR Green PCR master mix (Genstar) on the QuantStudio 6 Flex System (Applied Biosystems) according to the manufacturer’s instructions. The specific primers used for each gene are given in Table S1. The expression of each target gene was normalized using the *ACTIN* gene (LOC_Os03g50885). All experiments were carried out three times.

### Subcellular Localization

The *p*3*5S:OsNBL1-GFP* construct was co-expressed with RFP-HDEL (ER marker) or Man1-RFP (Golgi marker) in rice protoplasts by PEG-mediated transformation. Fluorescence signals were detected after the transformed protoplasts were incubated at 28 °C for 14 h under low-light conditions. The *p*3*5S:OsNBL1-GFP* vector was also co-expressed with RFP-SKL (peroxisome marker) in *Nicotiana benthamiana* leaves mediated by *A. tumefaciens*, and fluorescence was detected after 72 h infiltration. GFP (excitation wavelength: 488 nm, emission wavelength: 490 to 542 nm) and RFP (excitation wavelength: 561 nm, emission wavelength: 588 to 648 nm) fluorescent signals were examined and photographed using a laser confocal scanning microscope (Leica TCS SP5) according to the manufacturer’s instructions.

### Y2H Screening Assay

Y2H screening of the interaction proteins of OsNBL1 was performed using a mating method as described in the user manual (Clontech). The fragment sequence of 1 to 210 bp for *OsNBL1,* without the transmembrane domain coding sequence, was amplified and inserted into pGBKT7 to generate the bait construct BD-OsNBL1. The BD-OsNBL1 vector was transformed into yeast Y2H gold cells and mated with Y187 yeast containing the rice cDNA library. Positive clones were selected by culturing on quadruple-dropout (QDO) medium (SD/−Leu/−Trp/−His/−Ade).

The AD-OsClp6 vector was constructed by amplifying the full-length coding sequence of OsClp6, which was then cloned into the pGADT7 vector. The interaction between BD-OsNBL1 and AD-OsClp6 in yeast was analyzed by the co-transformation method according to the yeast protocols handbook (Clontech). The sequence of primers used for generating the constructs are listed in Table S1.

### LCI Assay

Full-length coding sequences of OsNBL1 or OsClp6 were cloned into LUC vectors to generate cLUC-OsNBL1 or OsClp6-nLUC constructs. cLUC-OsNBL1 and OsClp6-nLUC constructs were transformed into *Nicotiana benthamiana* leaf cells via *Agrobacterium*-mediated transformation, and luminescence was detected after 48 h of infiltration with a cooled CDD apparatus (Tanon 5200). Details of the operation were described in a previous report (Chen et al., 2008). Sequences of primers used in this study are listed in Table S1.

### Statistical Analysis

Data of each bar chart are presented as the mean ± SD. Microsoft Excel 2019 was used for bar charts construction and statistical testing. Significance analysis of the data was completed by Student’s *t*-test (*P < 0.05, **P < 0.01).

## Supplementary Information

**Additional file 2: Fig. S1.** Agronomic trait of *nbl1* and WT plants. **Fig. S2.** Detection of ROS accumulation in *nbl1* and WT plants. **Fig. S3.** Expression of defense-related genes in *nbl1* and WT plants. **Fig. S4.** Response to exogenous SA, MeJA, ACC and ABA. **Fig. S5.** Molecular cloning and analysis of the *OsNBL1* gene. **Fig. S6.** Phenotype of WT, *nbl1*, Com-1, Com-2 and Com-3 plants. **Fig. S7.** The expression of *OsNBL1* in WT, *nbl1*, and five OE plants. **Fig. S8.** Subcellular localization of OsNBL1 in the *N. benthamiana* leaves and rice protoplasts. **Fig. S9.** Interaction proteins of OsNBL1 identified by Yeast two hybrid screening. **Table S1.** List of primers used in this study. **Table S2.** Genetic analysis of *nbl1* mutant.

## Data Availability

All data generated or analyzed during this study are included in this article (and its supplementary information files).

## Abbreviations

CDS: Coding sequence
DAB: 3,3′-diaminobenzidine
DDI: Days after dark incubation
DINs: Dark-induced genes
ER: Endoplasmic reticulum
hpi: H post inoculation
LCI: Luciferase complementation imaging
MES: 2-(N-Morpholino)ethanesulfonic acid
NBT: Nitro blue
RT-qPCR: Real-time quantitative PCR
SAGs: Senescence-associate genes
TAIL-PCR: Thermal Asymmetric Interlaced PCR
Y2H: Yeast two hybrid
