# Supplementary material for: OsNBL1, a Multi-Organelle Localized Protein, Plays Essential Roles in Rice Senescence, Disease Resistance, and Salt Tolerance
Source: Rice (N Y). 2021 Jan 9;14:10. doi: 10.1186/s12284-020-00450-z (PMC7797018; doi:10.1186/s12284-020-00450-z)
Supplement: Supplementary file 2 — Additional file 2: Fig. S1. Agronomic trait of nbl1 and WT plants. Fig. S2. Detection of ROS accumulation in nbl1 and WT plants. Fig. S3. Expression of defense-related genes in nbl1 and WT plants. Fig. S4. Response to exogenous SA, MeJA, ACC and ABA. Fig. S5. Molecular cloning and analysis of the OsNBL1 gene. Fig. S6. Phenotype of WT, nbl1, Com-1, Com-2 and Com-3 plants. Fig. S7. The expression of OsNBL1 in WT, nbl1, and five OE plants. Fig. S8. Subcellular localization of OsNBL1 in the N. benthamiana leaves and rice protoplasts. Fig. S9. Interaction proteins of OsNBL1 identified by Yeast two hybrid screening. Table S1. List of primers used in this study. Table S2. Genetic analysis of nbl1 mutant. [file 12284_2020_450_MOESM2_ESM.docx]

**Supplement Information**

Article title: OsNBL1, a multi-organelle localized protein, plays essential roles in rice senescence, disease resistance, and salt tolerance

Authors: Xiaosheng Zhao^1, 2^, Tianbo Zhang^1^, Huijing Feng^1^, Tiancheng Qiu^1^, Zichao Li^2^, Jun Yang^1^, You-Liang Peng^1^ and Wensheng Zhao^1^*

^1^ State Key Laboratory of Agrobiotechnology, MOA Key Lab of Pest Monitoring and Green Management, Department of Plant Pathology, China Agricultural University, Beijing 100193, China.

^2^ Key Laboratory of Crop Heterosis and Utilization, the Ministry of Education/Key Laboratory of Crop Genetic Improvement, Beijing Municipality/ College of Agronomy and Biotechnology, China Agricultural University, Beijing 100193, China

*** For correspondence:**

Wensheng Zhao

Email: [mppzhaws@cau.edu.cn](mailto:mppzhaws@cau.edu.cn)

Tel: 86(10)62732541.

**
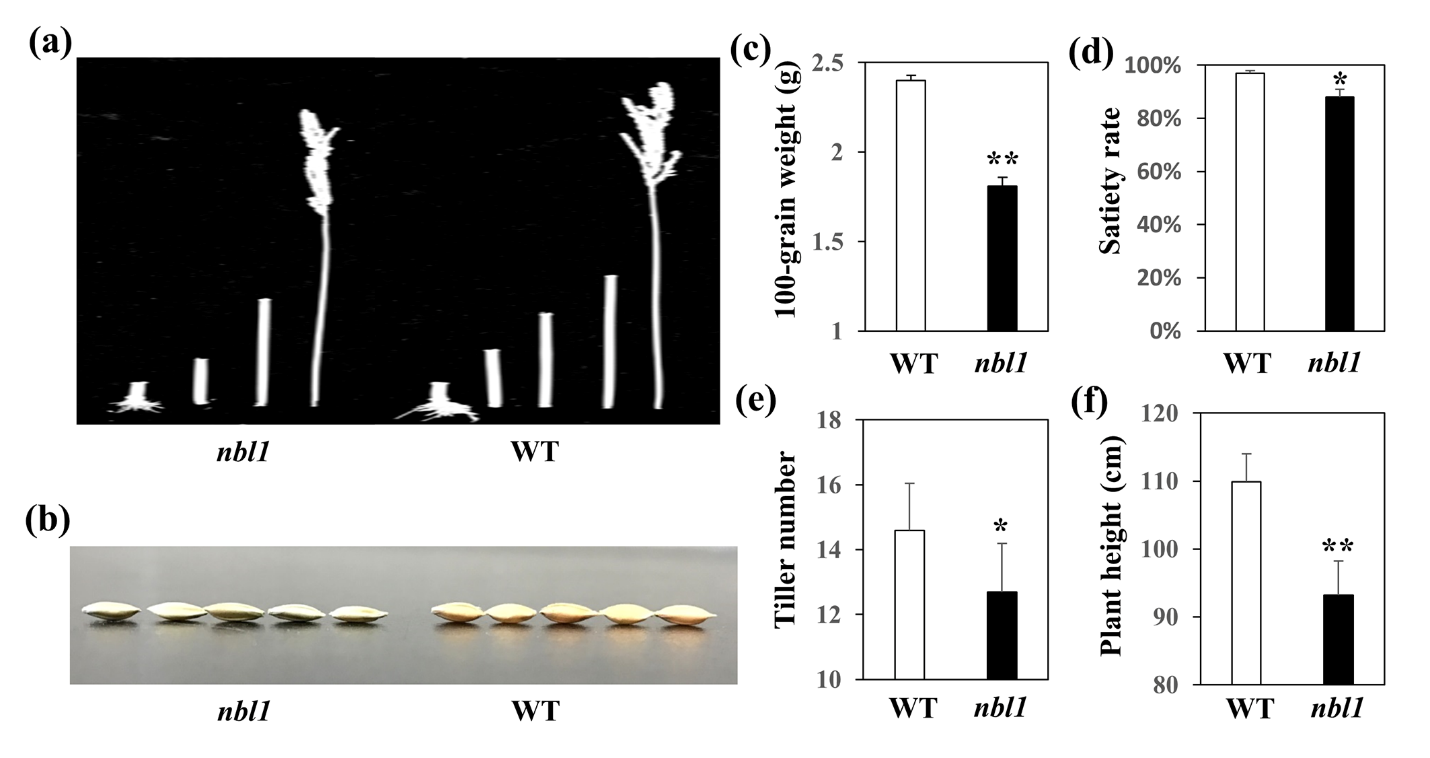
**

**Figure S1.** **Agronomic trait of *nbl1* and WT plants.**

(a-b) Pictures of internodes and seeds from *nbl1* and WT plants. Compared with WT plants, *nbl1* mutant lacks one internode and shows shriveled grain.

(c-f) *nbl1* mutant displays reduced 100 grain weight (c), satiety rate (d), tiller number (e) and plant height (f) compared with WT plants.


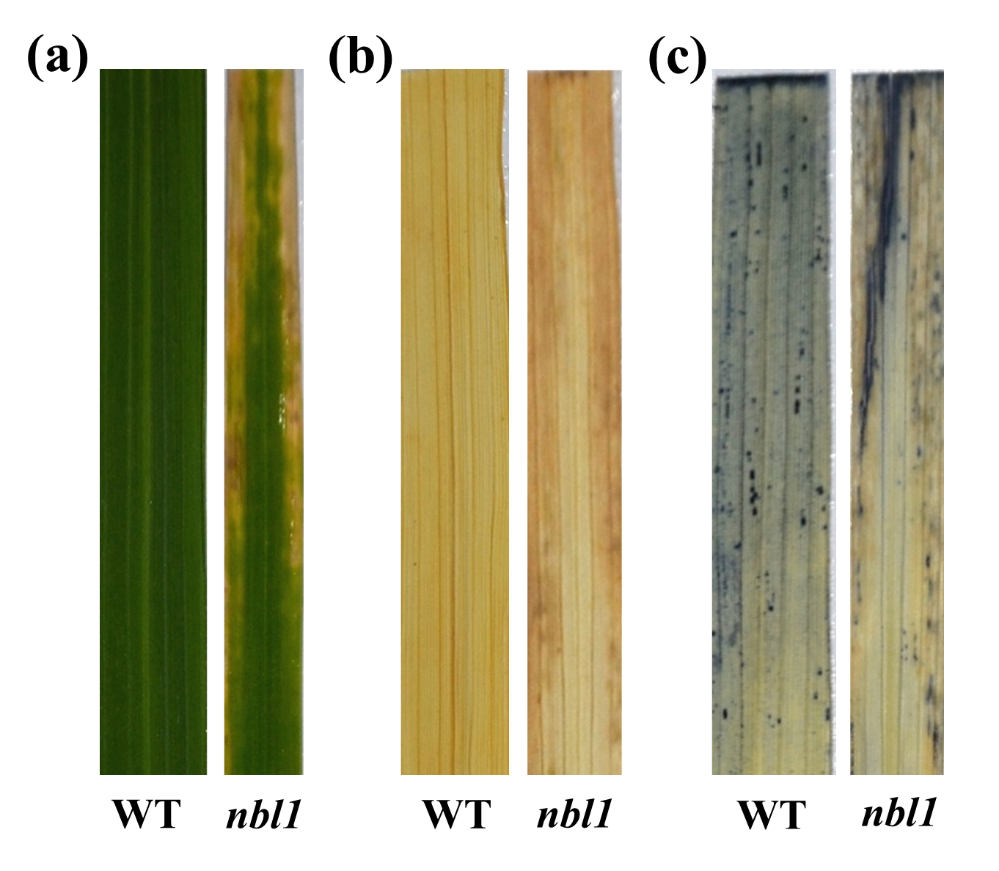


**Figure S2.** **Detection of ROS accumulation in *nbl1* and WT plants.**

(a) Phenotype of flag leaves of *nbl1* and WT plants grown under poddy filed condition at heading stage.

(b-c) DAB stanning and NBT stanning analysis of flag leaves of *nbl1* and WT plants shown in Figure S2 (a).

**
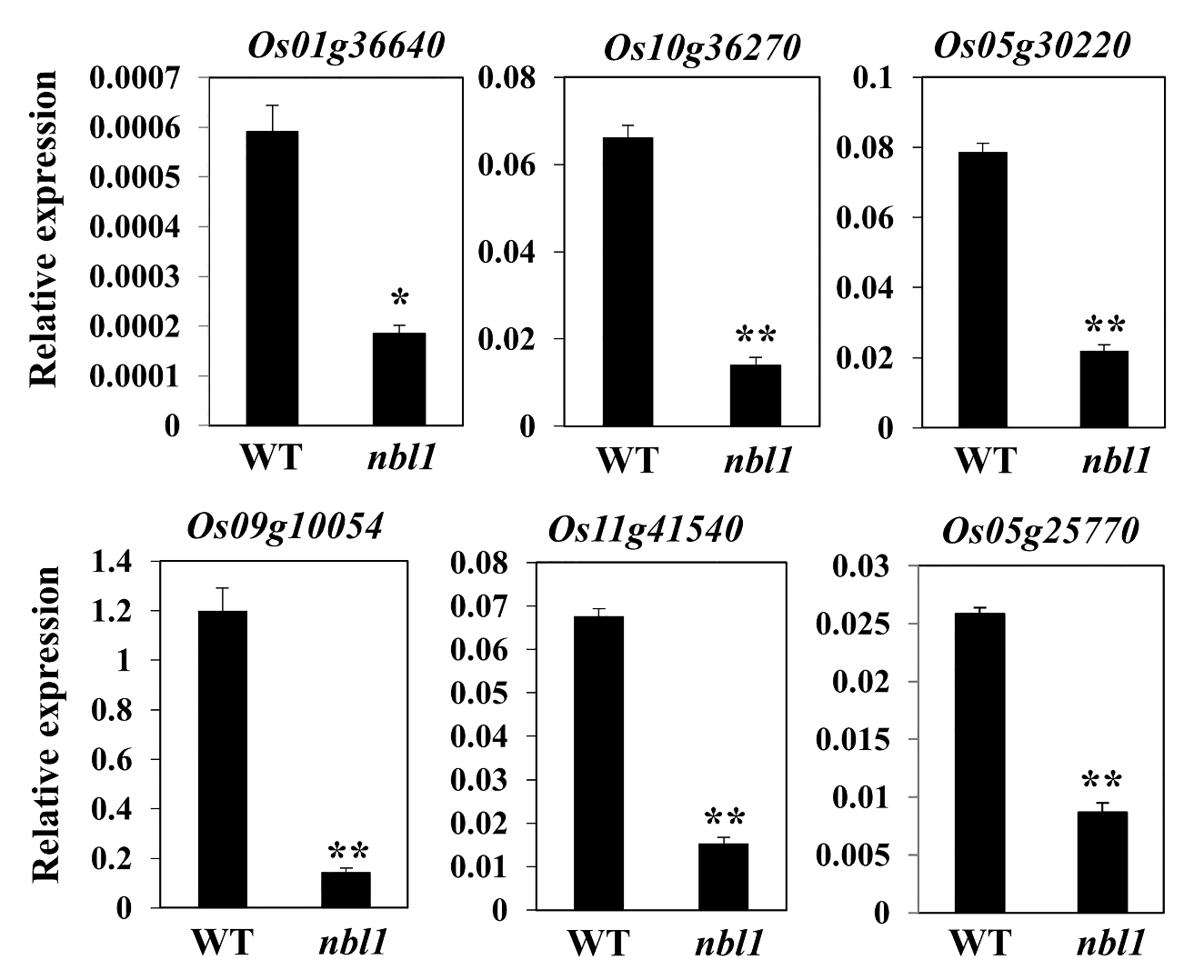
**

**Figure S3.** **Expression of defense-related genes in *nbl1* and WT plants.**

RT-qPCR analysis of the transcription level of six defense-related genes in *nbl1* and WT plants. Total RNA was extracted from the rice leaves at seedling stage. Data were normalized to the expression of the *OsACTIN* gene. Values are means ± SD (n=3). ***P* < 0.01 (Student’s *t*-test).


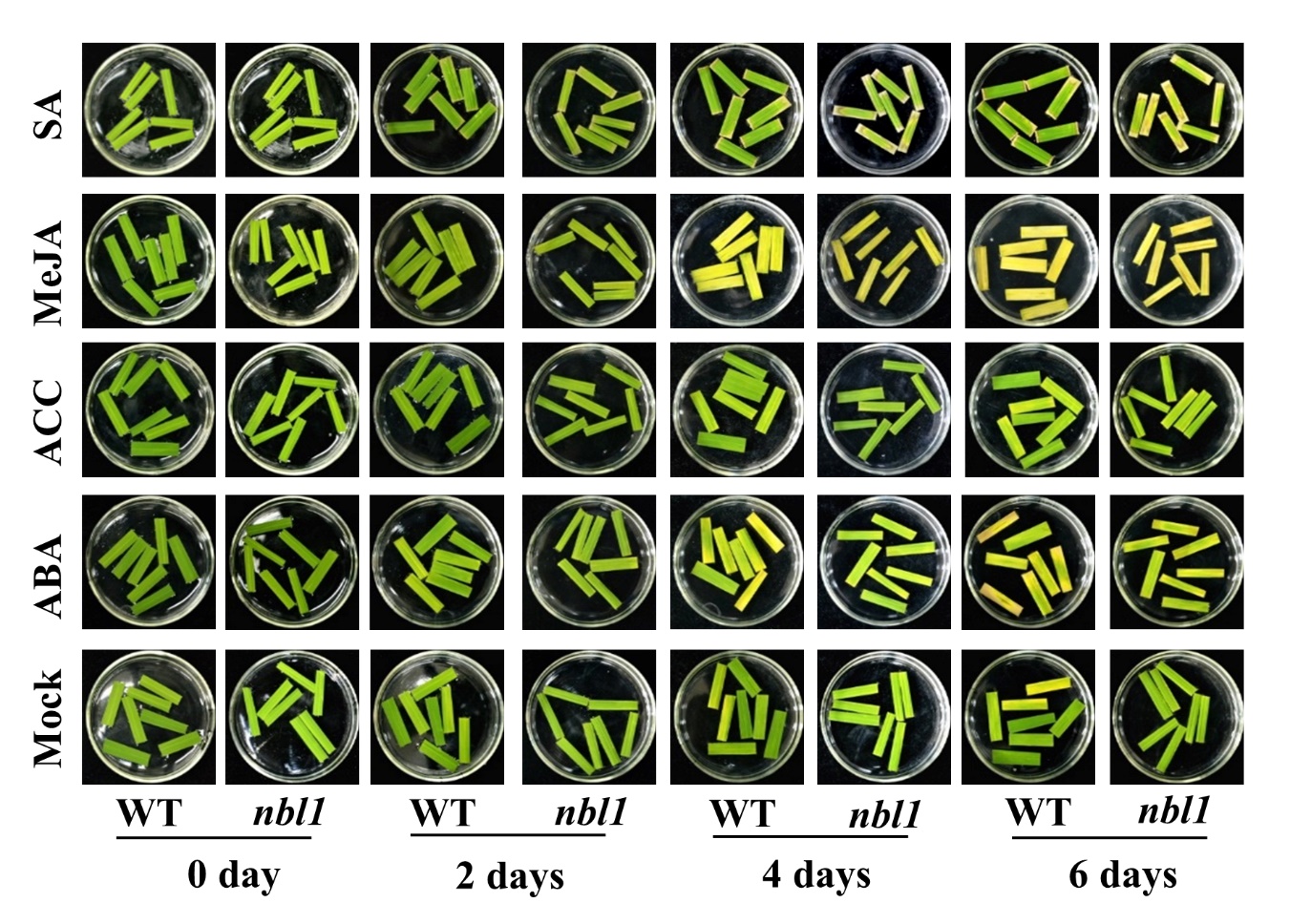


**Figure S4.** **Response to exogenous SA, MeJA, ACC and ABA.**

Detached leaves of *nbl1* and wild-type plants grown for six-weeks-old seedlings were immersed in buffer solution (3mM MES, pH 5.8) containing 100 μM SA, 100 μM MeJA, 10 mM ACC and 50 μM ABA at room temperature with 24 h light, respectively. Photographed at the indicated times.


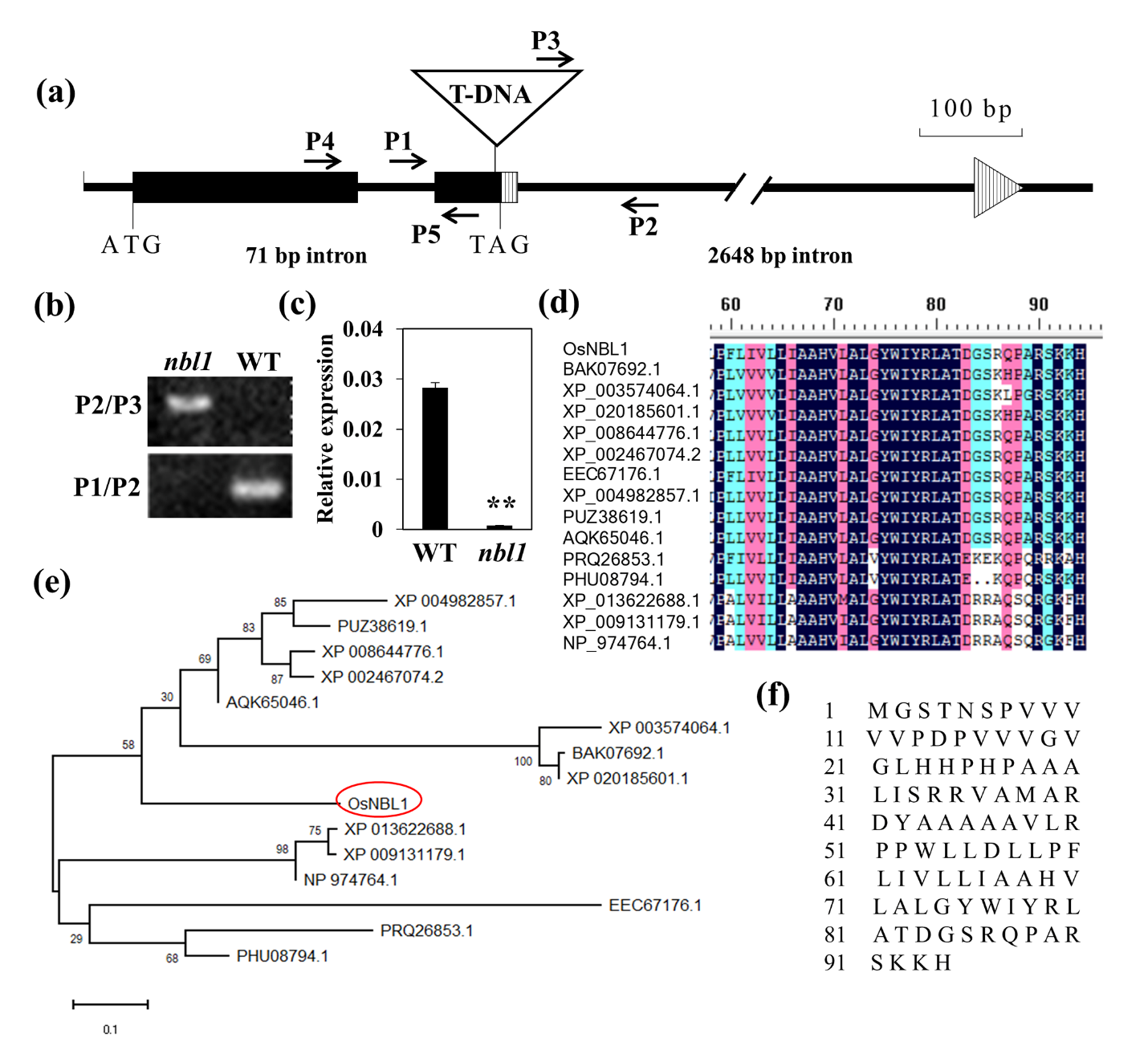


**Figure S5.** **Molecular cloning and analysis of the *OsNBL1* gene.**

(a) Schematic diagram indicating the T-DNA was inserted into the second exon of *OsNBL1*. Three primers, P1, P2 and P3, were used for identifying the T-DNA insertion in the nbl1 mutant. P4 and P5 are a pair of primers used to RT-qPCR analysis of the expression of *OsNBL1*.

(b) The T-DNA insertion site was reconfirmed by special PCR. Primer P1, P2 and P3 are shown in Figure 6(A).

(c) RT-qPCR analysis of the expression of *OsNBL1* in the leaves from *nbl1* and WT plants at seedling stage. Data were normalized to the expression of the *OsACTIN1* gene. Values are means ± SD (n=3). ***P* < 0.01 (Student’s *t*-test).

(d-e) Protein alignment (d) and phylogenetic analysis (e) of OsNBL1 and its 14 homologs from monocot and dicot plants. Sequence alignment was performed using ClustalW software. The phylogenetic tree was constructed by MEGA X using the neighbor-joining method. Numbers at nodes are bootstrap values based on 1000 replicates. Scale bar represents 0.1 substitutions.

(f) *OsNBL1* encodes a protein with 94 amino acids.

**
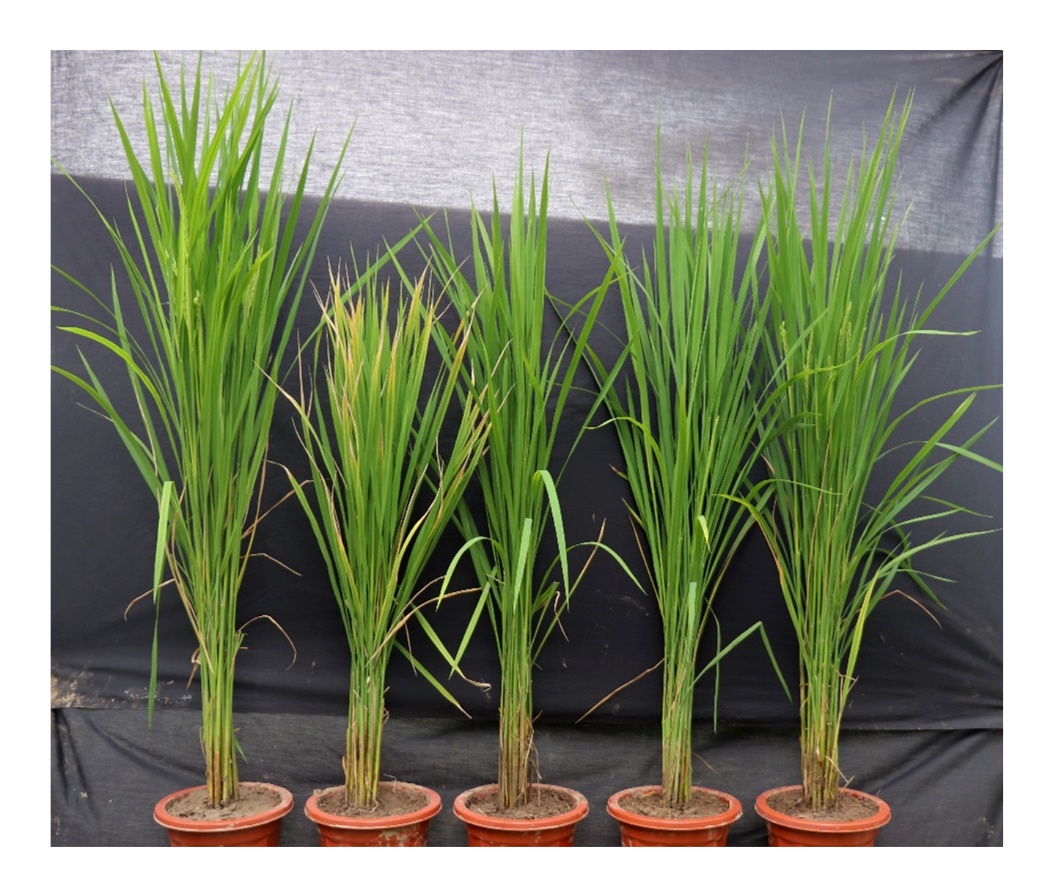
**

**Figure S6.** **Phenotype of WT, *nbl1*, Com-1, Com-2 and Com-3 plants.**

Picture of WT, *nbl1*, Com-1, Com-2 and Com-3 plants grown under poddy filed condition at heading stage. Com-1, Com-2 and Com-3 represent three complementary plants, which were generated by introducing *OsNBL1* into *nbl1* background.


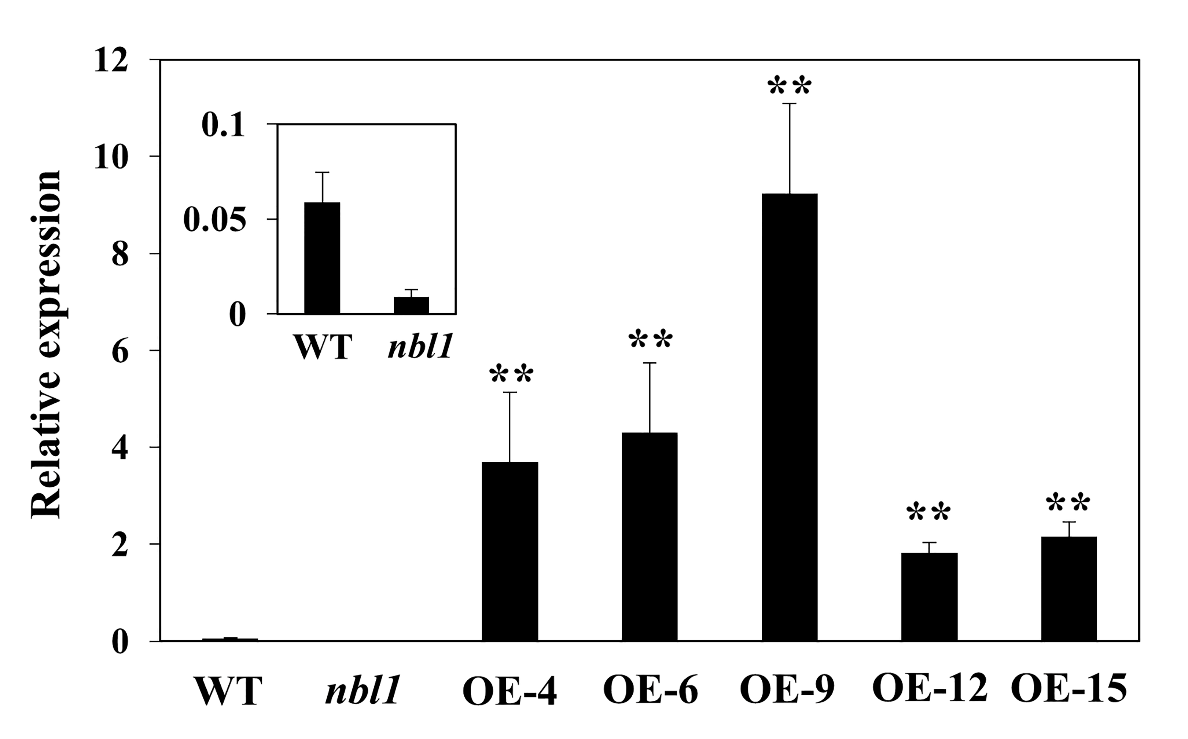


**Figure S7.** **The expression of *OsNBL1* in WT, *nbl1*, and five OE plants.**

RT-qPCR analysis of the transcription level of *OsNBL1* in WT, *nbl1*, OE-4, OE-6, OE-9, OE-12, and OE-15 plants. Total RNA was extracted from the rice leaves at seedling stage. Data were normalized to the expression of the *OsACTIN* gene. Values are means ± SD (n=3). ***P* < 0.01 (Student’s *t*-test).


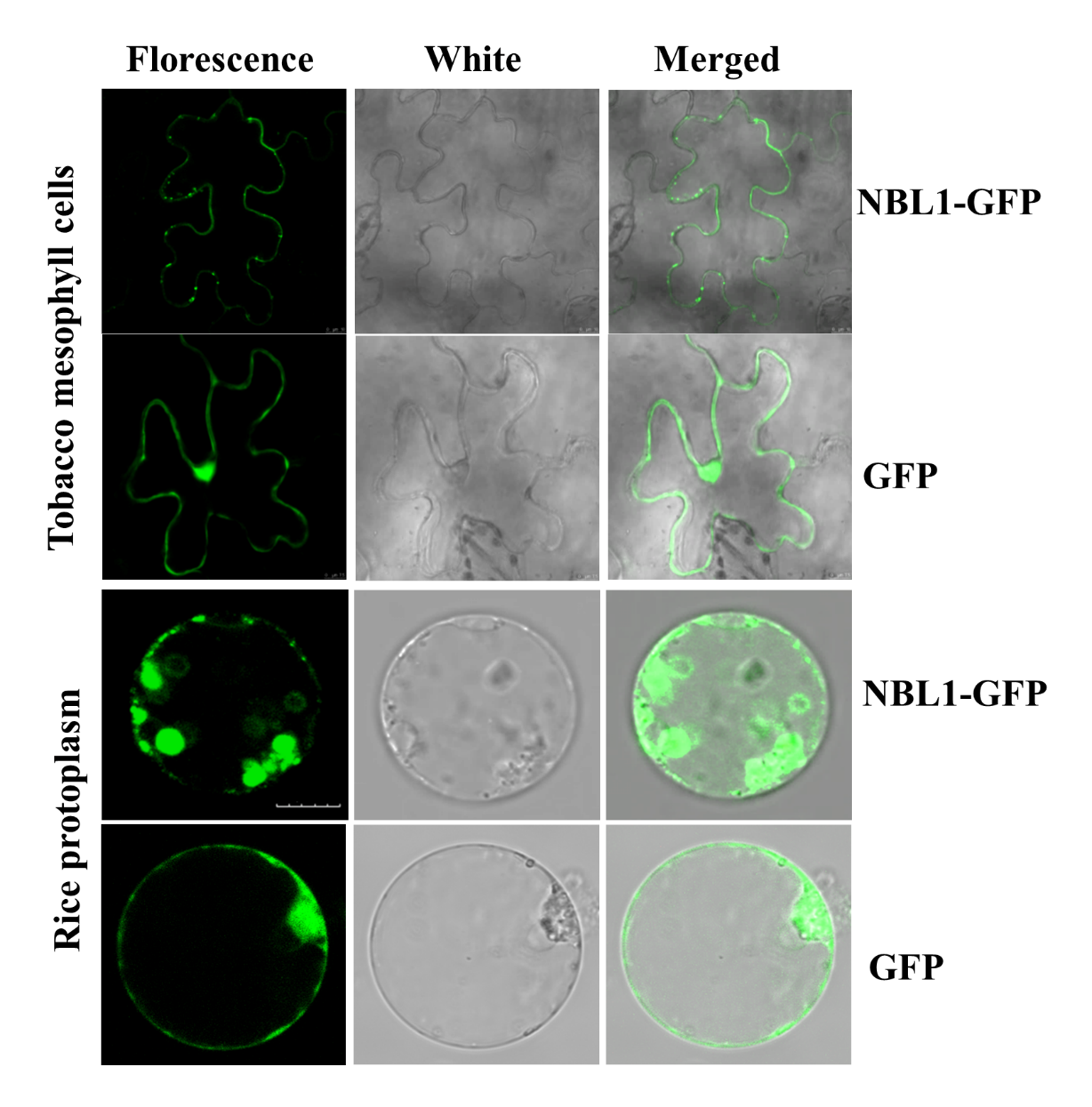


**Figure S8.** **Subcellular localization of** **OsNBL1 in the *N. benthamiana* leaves and rice protoplasts.**

OsNBL1-GFP construct or GFP control vector was transiently expressed in the *N. benthamiana* leaves (top panel) or rice protoplasts (bottom panel). GFP signal was monitored at 72 h or 14 h after transformation.

**
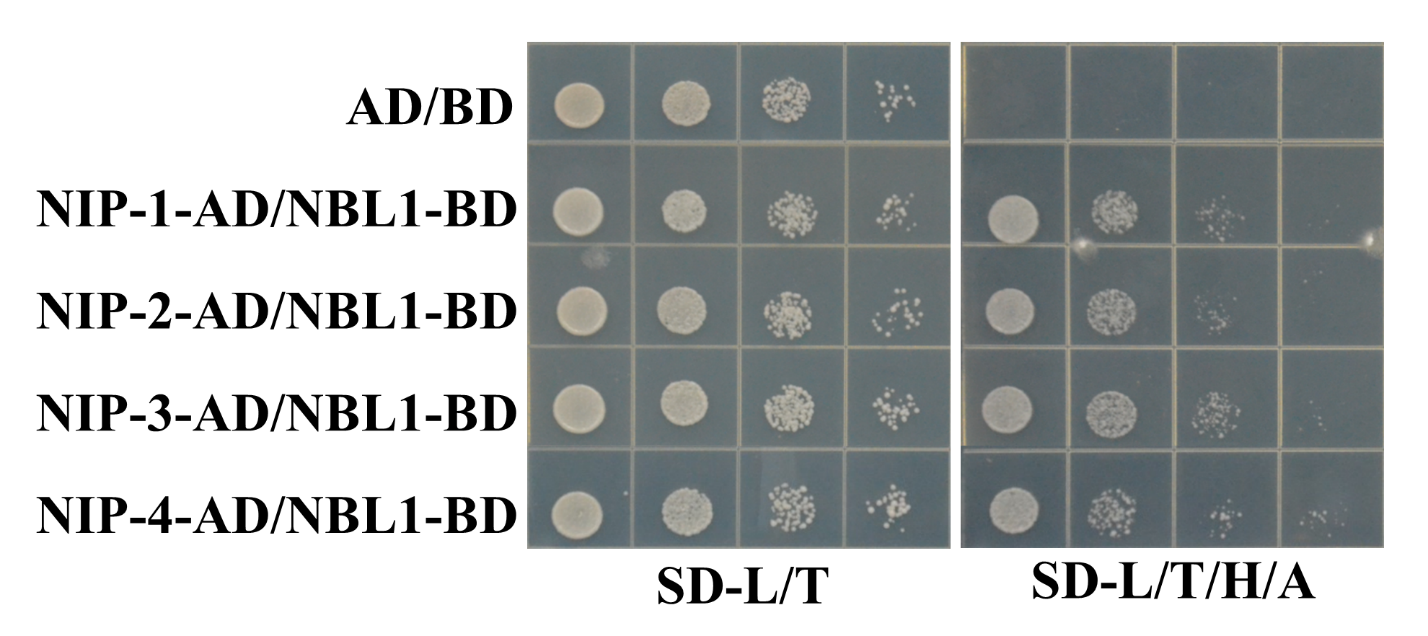
**

**Figure S9.** **Interaction proteins of OsNBL1 identified by Yeast two hybrid screening.**

Y2H shows that four candidate OsNBL1 Interaction Proteins (NIPs) screened from the yeast-two hybrid cDNA library of rice. OsNBL1-BD was as a bait.

**Table S1. List of primers used in this study.**

| Primer | Sequence of primer | Gene loc. |
| --- | --- | --- |
| qRT-OsActin-F | ATCACTGCCTTGGCTCCTA | LOC_Os03g50885 |
| qRT-OsActin-R | CATCTGCTGGAATGTGCTG |  |
| qRT-RCCR1-F | CGCATTTCCTCATGGAATTT | LOC_Os10g25030 |
| qRT-RCCR1-R | CTTCTCACGCTGTTTGTCCA |  |
| qRT-SGR-F | AGGGGTGGTACAACAAGCTG | LOC_Os09g36200 |
| qRT-SGR-R | GCTCCTTGCGGAAGATGTAG |  |
| qRT-OsI57-F | GCACGGAGGCGAACGA | LOC_Os01g52640 |
| qRT-OsI57-R | CTCCCAGCCTGCAAAGTTCATG |  |
| qRT-OsI85-F | GAGCAACGGCGTGGAGA | LOC_Os07g34520 |
| qRT-OsI85-R | GCGGCGGTAGAGGAGATG |  |
| qRT-OsNAP-F | CAAGAAGCCGAACGGTTC | LOC_Os03g21060 |
| qRT-OsNAP-R | GTTAGAGTGGAGCAGCAT |  |
| qRT-DIN1-F | GCTCTGCTGGTTTTACTGCTGTC | LOC_Os06g50930 |
| qRT-DIN1-R | TCACTTGTTGGTTGGGAGTTCATTC |  |
| qRT-DIN2-F | GCAGTGTTGGAGATTCAGGAAGG | LOC_Os04g39900 |
| qRT-DIN2-R | TTGGATTTGGAGCGGGAGAAGG |  |
| qRT-DIN3-F | CAAGTGGAGTTACTGGAGGGAAGG | LOC_Os01g21160 |
| qRT-DIN3-R | TATGAGGAATGGTAGGAAGGTGTGC |  |
| qRT-DIN4-F | TCACTACCATTCACAGTCACCAGAG | LOC_Os07g07470 |
| qRT-DIN4-R | GGAACTTCTTCAACAGCCAAACGG |  |
| qRT-DIN6-F | GGGCTGGGGTCTTGATGGTTC | LOC_Os06g15420 |
| qRT-DIN6-R | GGAAGGATATGAATCGCTCGCAATC |  |
| qRT-DIN9-F | GCTCACTACCACCAGGCGAAC | LOC_Os01g03710 |
| qRT-DIN9-R | GGACCAGAGGCAGAGAACTTAACC |  |
| qRT-HAK1-F | GTTGATGATGCTGATGTTGGAAG | LOC_Os09g39110 |
| qRT-HAK1-R | CCAACACTTTCAGCTGAAAC |  |
| qRT-HAK5-F | CATTGTGGACTATTTGAAAGAA | LOC_Os01g70490 |
| qRT-HAK5-R | GGAGAACTACAGAAAAGCCAATC |  |
| qRT-SNAC1-F | TTGGGATCAAGAAGGCGCTC | LOC_Os03g60080 |
| qRT-SNAC1-R | GCACCCCAATCATCCAACCT |  |
| qRT-NAC3-F | GCTGGATGACTGGGTGCTGTG | LOC_Os07g37920 |
| qRT-NAC3-R | CCTCCTCCTCCTTCCTGCTCTG |  |
| qRT-NAC10-F | CCTAATATACACAACACCTCATCCA | LOC_Os11g03300 |
| qRT-NAC10-R | GTCATTGCTGCTGCCATC |  |
| qRT-LEA3-1-F | GGCGAGTGAGCAGGTGAAGAG | LOC_Os05g46480 |
| qRT-LEA3-1-R | GCGGTGGCAGAGGTGTCC |  |
| qRT-RPM1-F | AACAGTAGAGGAAGTGGCAGAGG | LOC_Os01g36640 |
| qRT-RPM1-R | TGAGGGCAAGTAAACGGATTATGTC |  |
| qRT-RPP13-F | CCGTTTTCTGCGACACCTTGAG | LOC_Os10g36270 |
| qRT-RPP13-R | AGGCGTTGGAGACCGTTGG |  |
| qRT-RP1L1-F | GCCTCCTCCATCGGTTCCATC | LOC_Os05g30220 |
| qRT-RP1L1-R | TTATGTTCTTCGGTCGCAGATTGG |  |
| qRT-RPS2-F | ATTCCCATCCCTAAAGAGCCTGAG | LOC_Os09g10054 |
| qRT-RPS2-R | TTATCCCACCATTCAACACTATCGG |  |
| qRT-RPP8-F | TCTATATCTACGGCGGACCTTGTTG | LOC_Os11g41540 |
| qRT-RPP8-R | ACTACTTGGACGAGGCTTCTTTGAG |  |
| qRT-WRKY45-F | ACCAGATTCTCCACACCTTCTCC | LOC_Os05g25770 |
| qRT-WRKY45-R | GCACCTCCGACCCTTCTCC |  |
| P1 | AGCGCTTAGACCAACAAGAGA |  |
| P2 | ATTCGGCCTAAAAATCCCTCTC |  |
| P3 | CTACGGCTGGCCTAGACAGCTAG |  |
| qRT-NBL1-F | CCTTCCTCATCGTCCTCCTCATC | LOC_Os10g33855 |
| qRT-NBL1-R | TGGCGAGCCTGTAGATCCAG |  |
| OE-NBL1-F | ATAAAGCTTATGGGCTCGACGAACAG |  |
| OE-NBL1-R | TATACTAGTCTAGTGCTTCTTGCTC |  |
| GFP-NBL1-F | ATAGGTACCATGGGCTCGACGAACAG |  |
| GFP-NBL1-R | ATAGTCGACATAGTGCTTCTTGCTCC |  |
| BD-NBL1-F | ATAGAATTCATGGGCTCGACGAACAG |  |
| BD-NBL1-R | TATCTGCAGCTAGTGCTTCTTGCTC |  |
| cLUC-NBL1-F | ATAGGTACCATGGGCTCGACGAACAG |  |
| cLUC-NBL1-R | TATGTCGACCTAGTGCTTCTTGCTCC |  |
| AD-ClpP6-F | ATAGAATTCATGGCGCCTATGGCC | LOC_Os03g29810 |
| AD-ClpP6-R | ATAGGATCCTTAGTATCTTGTTTCCAGC |  |
| nLUC-ClpP6-F | ATAGGTACCATGGCGCCTATGGCC |  |
| nLUC-ClpP6-R | TATGTCGACGTATCTTGTTTCCAGCAG |  |

**Table S2. Genetic analysis of *nbl1* mutant**

| Progenies | Total | Wild-type phenotype | Mutant phenotype | χ^2^_0.05,1_=3.84, (3:1) |
| --- | --- | --- | --- | --- |
| T1 generation | 16 | 12 | 3 | 0.18 |
| F2 population of Nipponbare/*nbl1* | 145 | 106 | 37 | 0.28 |
